# Assembly of Diverse 3-Methylenetetrahydrofurans via Palladium-Catalyzed Cascade Carboetherification of Buta-1,3-Dienes with Alkynols

**DOI:** 10.3390/molecules30214244

**Published:** 2025-10-31

**Authors:** Yin-Long Lai, Yanan Niu, Kaitong Zeng, Huanfeng Jiang, Huishi Guo, Sheng-Ling Zhang, Jianxiao Li

**Affiliations:** 1 College of Chemistry and Civil Engineering, Shaoguan University, Shaoguan 512005, China; 2 Guangdong Provincial Engineering Technology Research Center for Eco-Environmental Protection and Solid Waste Utilization in Northern Guangdong, Shaoguan University, Shaoguan 512005, China; 3 Key Laboratory of Functional Molecular Engineering of Guangdong Province, School of Chemistry and Chemical Engineering, South China University of Technology, Guangzhou 510640, China; 4 Guangdong Provincial Key Laboratory of Utilization and Conservation of Food and Medicinal Resources in Northern Region, Shaoguan University, Shaoguan 512005, China

**Keywords:** cascade cyclization, palladium catalysis, carboetherification, tetrahydrofuran heterocyclics, alkynes

## Abstract

Tetrahydrofuran scaffolds have found promising applications in both pharmaceutical chemistry and organic synthesis but remain underexplored owing to the challenges associated with their preparation. Herein, we have developed a robust synthetic strategy for the palladium-catalyzed cascade annulation reaction of buta-1,3-dienes with alkynols for the assembly of diverse 3-methylenetetrahydrofurans with excellent functional group compatibility and good regioselectivity. More importantly, this protocol features broad substrate scope, good functional group tolerance, and good step- and atom-economy. A diverse array of functional groups such as halogen atoms, aldehyde, nitro, ester, and several aromatic heterocycles were also nicely tolerated and provided the corresponding products in moderate to good yields. It is noteworthy that the practicability is further verified by gram-scale experiments and synthetic transformation to access organic functional molecules.

## 1. Introduction

**Figure 1:**
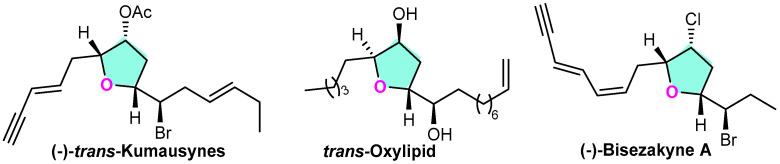
Examples of biologically active tetrahydrofuran derivatives.

As an important branch of heterocyclic frameworks, oxygen-containing five-membered heterocyclic compounds such as tetrahydrofuran architectures have proven to be excellent scaffolds for the assembly of structurally diverse pharmaceutical and bioactive molecules [1,2,3]. As depicted in Figure 1, the (−)-*trans*-Kumausyne was isolated from the red alga Laurencia nipponica and further used as an antibiotic drug [4]. Moreover, *trans*-Oxylipid was reported as the inhibitor in parasitic nematodes in vitro [5]. In particular, the natural product (−)-Bisezakyne A, a halogenated C_15_-acetogenin, was isolated from the red algal genus *Laurencia* by Suzuki and co-workers in 1999 [6]. It is a remarkable fact that the integration of polysubstituted tetrahydrofuran moiety as the core structure into drug candidates may greatly enhance their biological activities [7]. As a result, the development of a practical and expeditious synthetic approach for the construction of polyfunctionalized tetrahydrofuran moieties using inexpensive and easily available raw materials is still highly desirable.

**Scheme 1:**
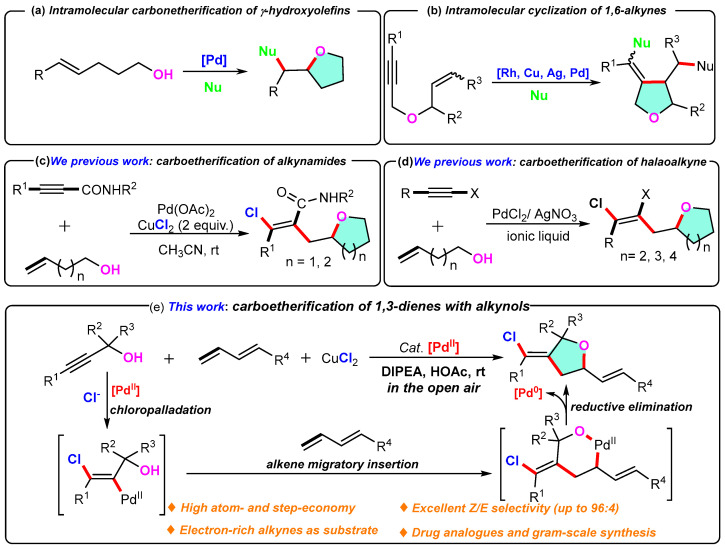
Strategies for the synthesis of tetrahydrofuran derivatives.

Given the importance of the five-membered heterocyclic building blocks, numerous synthetic approaches have been successfully exploited for the construction of these diverse structural skeletons in recent years. In this regard, Semmelhack and co-workers reported the first intramolecular alkoxypalladation/carbonylation of enol for constructing 5- or 6-membered oxygen-containing heterocyclic compounds (Scheme 1a) [8]. However, the regioselectivity of alkene was uncontrollable under the developed catalytic system. After that, transition-metal-catalyzed intramolecular cascade cyclization/functionalization reactions of 1,6-enynes emerged as a flourishing strategy for the synthesis of various high-added-value heterocyclic scaffolds (Scheme 1b) [9]. Among them, palladium-catalyzed protocols have been widely investigated in this field, owing to their good functional group tolerance and high regio- and stereoselectivity [10,11,12,13,14]. For example, Chegondi and colleagues developed an elegant palladium-catalyzed regioselective cascade cyclization of cyclohexadienones-containing 1,6-enynes to afford a variety of *cis*-fused bicyclic frameworks containing a tetrahydrofuran motif with excellent regio- and stereoselectivity [13]. Furthermore, other transition metal catalysts, such as Rh [15,16,17,18], Ru [19,20], Co [21,22,23,24], and others [25,26,27,28,29], have also been identified as the most effective catalysts for assembling more elaborated tetrahydrofuran scaffolds. For instance, Tian, Lin, and co-workers demonstrated a rhodium-catalyzed cross-addition of cyclohexadienone-tethered internal alkynes to an assembly of the *cis*-hydrobenzofuran frameworks, with good yields [15]. Then, RajanBabu and co-workers realized an extraordinary cobalt-catalyzed ligand-controlled regioselective tandem cycloisomerization-hydroarylation and cycloisomerization-hydroalkenylation of 1,6-enynes [24]. In 2014, we developed a palladium-catalyzed hydroxyl chelation-assisted carboetherification of alkynamides with olefins for the synthesis of oxygenated heterocycles (Scheme 1c) [30]. In 2017, we also developed a palladium-catalyzed cascade carboetherification of haloalkynes with enols for the straightforward preparation of four- to seven-membered functionalized oxetanes (Scheme 1d) [31]. To the best of our knowledge, there is no previous synthetic method for the synthesis of a polyfunctionalized tetrahydrofuran structural motif by palladium-catalyzed cascade cyclization reactions from readily available alkynol with alkenes. In continuation of our interests in palladium-catalyzed cross-coupling of alkynes [32,33,34,35] and of our previous studies on functionalized heteroaromatic motifs [36,37,38], we herein disclose an efficient and practical palladium-catalyzed cascade protocol for a tunable assembly of diversified tetrahydrofuran architectures (Scheme 1e).

## 2. Results and Discussion

Our investigation was initiated by using 4-phenylbut-3-yn-2-ol (**1a**) and buta-1,3-dien-1-ylbenzene (**2a**) as the model substrates to optimize this cascade cyclization approach. As outlined in Table 1, the desired product **3a** was obtained in 86% GC yield (82% isolated yield) when this protocol was performed in the presence of 10 mol% of Pd(TFA)_2_, 4 equiv. of CuCl_2_, and 80 mol% of DIPEA in HOAc at room temperature for 24 h in open air (Table 1, entry 1). It is worth mentioning that the CuCl_2_ as the oxidant and chloride ion source was previously established for these *trans*-chloropalladation initiated cascade transformations of alkynes [39,40]. Various palladium catalysts were firstly screened, and Pd(TFA)_2_ proved to be optimal, producing the target product **3a** in 86% GC yield, while the other palladium sources, such as PdCl_2_, Pd(OAc)_2_, Pd(Py)_2_Cl_2_, and Pd(MeCN)_2_Cl_2_, gave inferior results (Table 1, entries 1–5). Subsequently, several solvents, including DMSO, MeCN, DCE, acetone, and HOAc, were then examined, and the results suggested that HOAc as the solvent was the best choice in this transformation (Table 1, entries 6–9). Different bases were also evaluated, and we found that DIPEA was a suitable base in this cascade approach (Table 1, entries 10–13). In addition, the increase of the temperature from r.t. to 40 °C led to a diminishing yield and the ratio of Z/E (Table 1, entry 14). Control experiments indicated that both Pd(TFA)_2_ and CuCl_2_ played a vital role in this transformation (Table 1, entry 15).

**Figure 2:**
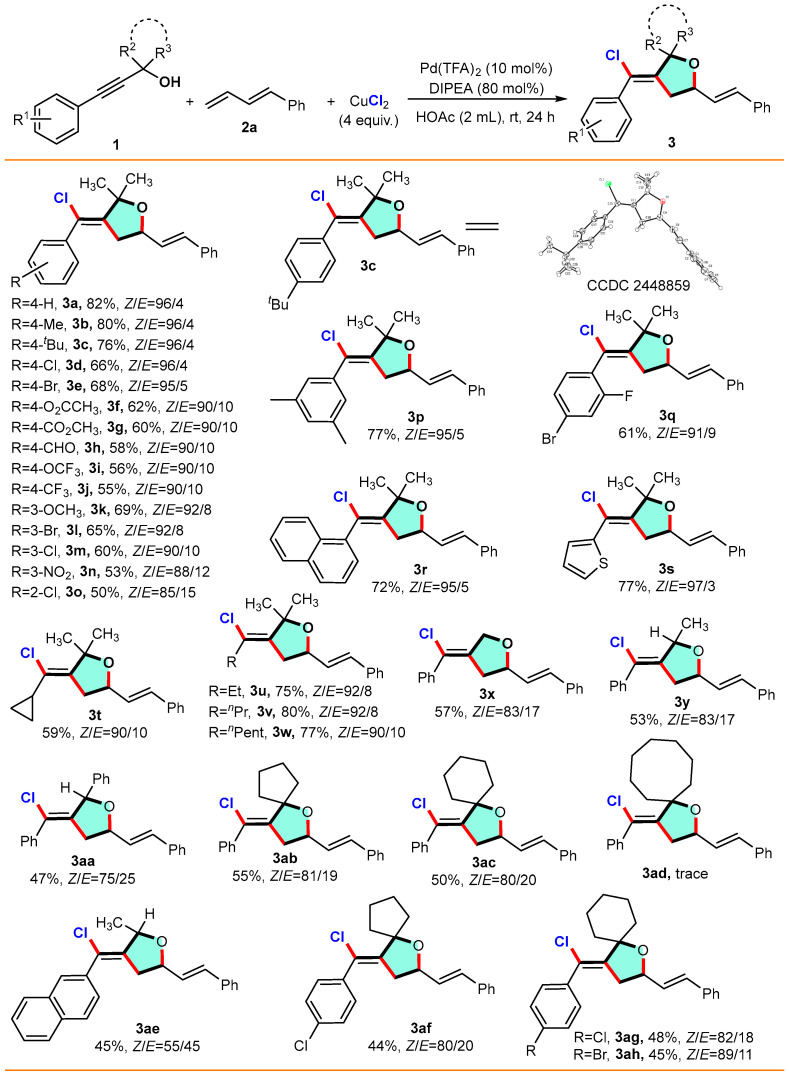
Substrate scope of alkynols. All reactions were performed with **1** (0.20 mmol), **2a** (0.30 mmol), Pd(TFA)_2_ (10 mol%), CuCl_2_ (4 equiv.), DIPEA (80 mol%), and HOAc (2 mL) stirred at room temperature for 24 h. Yields refer to isolated yield. The Z/E ratio was determined by GC-MS analysis of the crude material.

With the optimal reaction conditions in hand, we next examined the substrate scope of this cascade carboetherification of alkynols, and several representative examples are summarized in Figure 2. Gratifyingly, both electron-donating (Me, *^t^*Bu, OMe, OCF_3_) and electron-withdrawing (F, Cl, Br, CO_2_Me, CF_3_, CHO, NO_2_) substituents on the phenyl ring of alkynols were nicely amenable for these catalytic systems and generated the corresponding products **3a**–**3q** with yields ranging from 50% to 82% with high regioselectivities. Additionally, alkynol substrates with halogen atoms such as Cl and Br at the *ortho*-, *meta*-, and *para*-position had little influence on the reaction, furnishing the desired products **3d**, **3e**, and **3l**, **3m** in similar yields. The configuration of **3c** (CCDC 2448859) was determined by X-ray analysis (see Supplementary Materials). Notably, alkynol substrates with various sensitive functional groups, such as ester (**3g**), aldehyde (**3h**), trifluoromethyl (**3j**), and nitro (**3n**), were also well accommodated, offering the corresponding products in moderate to good yields. Delightfully, 2,4-, and 3,5-disubstituted alkynol substrates were perfectly compatible with our catalytic systems, giving the desired products **3p** and **3q** in 77% and 61% yields, respectively. Of particular interest is that the naphthyl and thienyl substituents were amenable to these reaction conditions, furnishing the corresponding products **3r** and **3s** in satisfactory yields. It is remarkable that alkyl alkynols also efficiently coupled with **2a** and gave the target products **3t**–**3w** in moderate to good yields. Of note, a great many structurally diverse alkynols, such as 3-phenylprop-2-yn-1-ol (**1x**), 4-phenylbut-3-yn-2-ol (**1y**), 1,3-diphenylprop-2-yn-1-ol (**1aa**), 1-(phenylethynyl)cyclopentan-1-ol (**1ab**), and 1-(phenylethynyl)cyclohexan-1-ol (**1ac**) can be applied as well in these catalytic systems, albeit in somewhat low yields.

**Figure 3:**
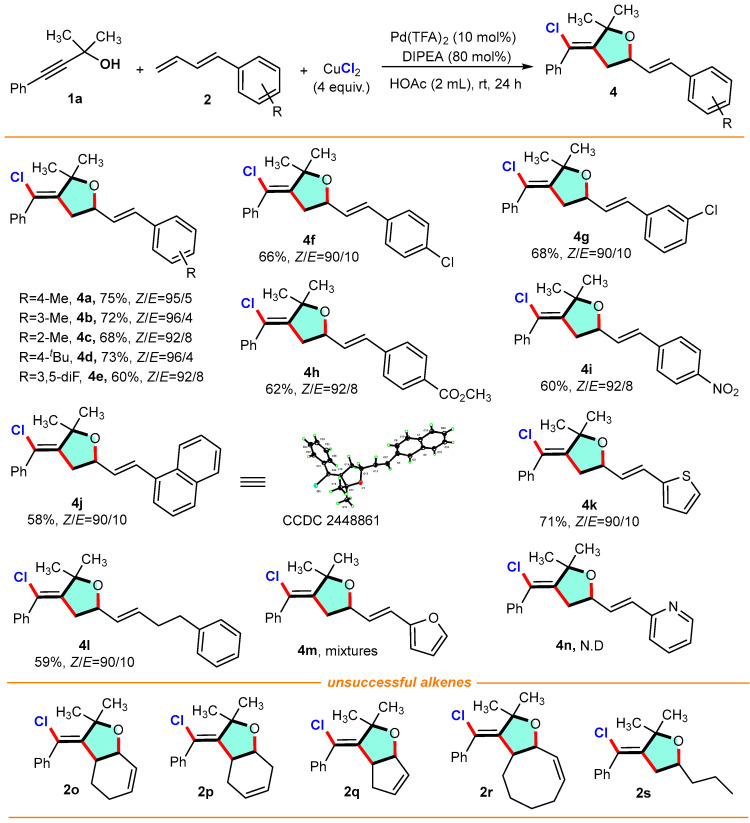
Substrate scope of alkenes. All reactions were performed with **1a** (0.20 mmol), **2** (0.30 mmol), Pd(TFA)_2_ (10 mol%), CuCl_2_ (4 equiv.), DIPEA (80 mol%), and HOAc (2 mL) stirred at room temperature for 24 h. Yields refer to isolated yield. The Z/E ratio was determined by GC-MS analysis of the crude material.

Inspired by the good results we obtained, we then evaluated the substrate generality of a diverse array of alkenes under our developed conditions. As outlined in Figure 3, the alkenes with a wide variety of functional groups, such as methyl, *tert*-butyl, fluorine, chlorine, ester, and nitro, on the benzene ring worked well in our catalytic systems, giving the desired products **4a**–**4d** in moderate to good yields (60–75%) and satisfactory Z/E ratio (96:4 to 90:10). Satisfactorily, the position of the substituent had little effect on the reaction outcomes, and the substrates with an *ortho*- or *meta*-substituent on the benzene ring gave similar outcomes of the desired products (**4a**–**4c**). Conjugated 1,3-dienes bearing a 1-naphthyl or 2-thienyl moiety were also amenable for these conditions and furnished the corresponding products **4j** and **4k** in 58% and 71% yields, respectively. The absolute configuration of **4j** was unambiguously determined by X-ray crystallography (see Supplementary Materials). Moreover, substrate hexa-3,5-dien-1-ylbenzene could also be efficiently transformed into the desired product **4l** in 59% yield. However, owing to the strong conjugative effect and coordination with the palladium catalyst, desired product **4n** was not detected by GC-MS analysis. Unfortunately, the substrates of cyclopenta-1,3-diene, cyclohexa-1,3-diene, cycloocta-1,3-diene, and hex-1-ene were unsuccessful in our developed catalytic systems and failed to furnish the corresponding products **4o**–**4s**.

**Scheme 2:**
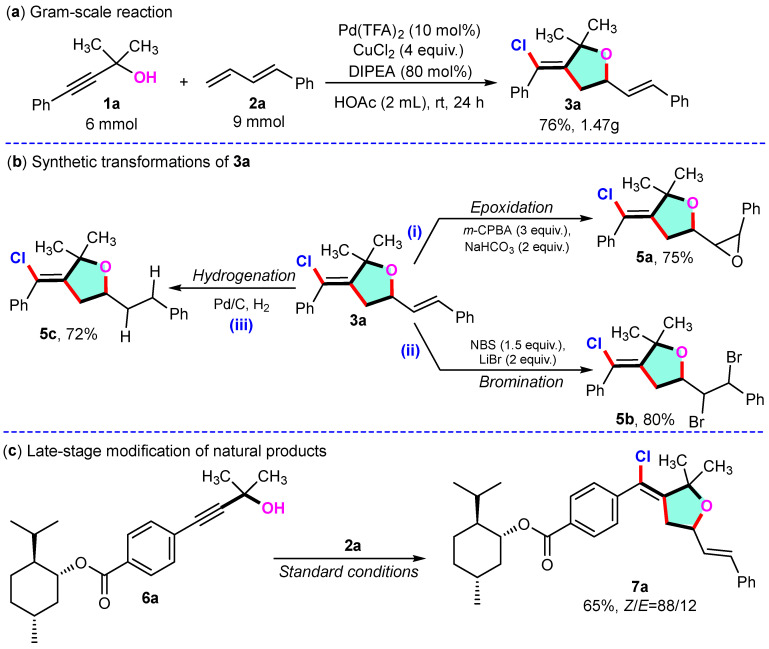
The gram-scale experiment and synthetic transformations.

To demonstrate the practicality of this cascade protocol, a gram-scale experiment and several synthetic transformations were carried out under our developed catalytic conditions. As shown in Scheme 2, when 6 mmol of **1a** was utilized, 1.47 g of the desired product **3a** was obtained in a 76% yield without erosion of the Z/E ratio (Scheme 2a). The carbon-carbon double bond offers a useful handle for further manipulation. Useful synthetic epoxides can be easily obtained through the epoxidation reaction of **3a**, giving the target product **5a** in a 75% yield [41]. In addition, in the presence of bromination reagents such as NBS and LiBr, the dibromination reaction was performed, and it delivered the corresponding product **5b** in an 80% yield [42]. Finally, the Pd/C reduction reaction of **3a** generated the corresponding **5c** in a 72% yield [43]. In addition, alkynol derived from a menthol natural product consistently furnished the corresponding cyclization product **7a** in a 65% yield (Scheme 2c).

**Scheme 3:**
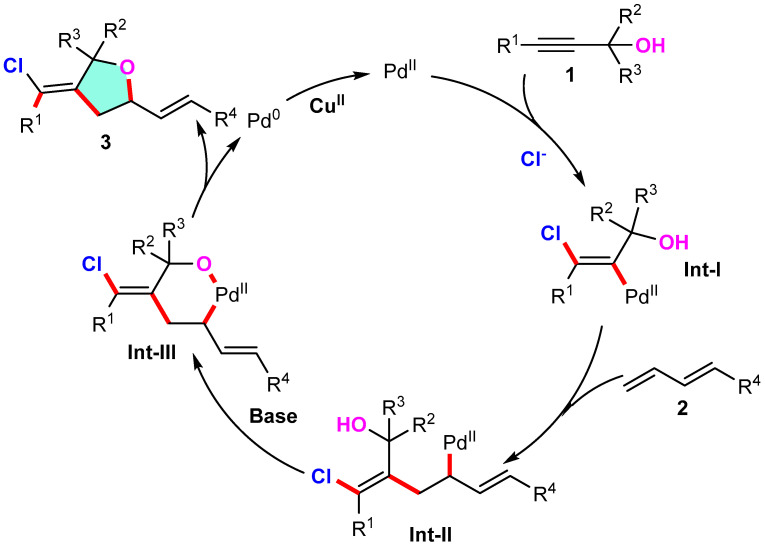
Plausible mechanistic pathways.

On the basis of the obtained results and precedents from the literature, we proposed a plausible mechanism for this palladium-catalyzed cascade carboetherification of buta-1,3-dienes with alkynols for the efficient synthesis of diverse 3-methylenetetrahydrofurans. As depicted in Scheme 3, in the presence of excess chloride ions [44,45], *trans*-chloropalladation of the alkynols gives the vinyl palladium species **int-I**. Then, the buta-1,3-dienes undergo migratory insertion to the palladium species **int-I**, generating σ-alkyl-Pd species **int-II** [46]. Subsequently, six-membered palladacycle species **int-III** is formed in the presence of the basic conditions. After that, a reductive elimination of the palladacycle **int-III** delivers the desired product **3** and palladium^(0)^ species. Finally, the oxidation of Pd^(0)^ species to Pd^(II)^ by Cu^(II)^ and air completes this catalytic cycle.

## 3. Materials and Methods

### 3.1. Materials

All the reagents were obtained from commercial sources and used directly without further purification. Melting points were measured on an Electrothemal SGW-X4 microscopy digital melting point apparatus (Wagner & Münz, Munich, Germany) and are uncorrected. ^1^H NMR and ^13^C NMR spectra were recorded on a Bruker AVANCE III 400 MHz (Bruker, Billerica, MA, USA) using CDCl_3_ as a solvent. The chemical shifts are referenced to signals at 0 or 7.26 and 77.0 ppm and chloroform as the solvent, with TMS as the internal standard. Chemical shifts (δ) are reported in ppm, and coupling constants (J) are reported in Hertz (Hz). ^19^F NMR spectra were collected at 376 MHz on the same instrument and are referenced to internal C_6_H_5_F at δ −113.15. GC-MS data were obtained with a Thermo Scientific TRACE (Milan, Italy) 1300 & ISQ QD system equipped with a TG-5MS column. High-resolution mass spectra (HRMS) were recorded on a JEOL JMS-600 spectrometer (Waters Corporation, Beijing, China). X-ray diffraction was measured on a ‘Bruker APEX-II CCD’ diffractometer (Bruker, Billerica, MA, USA) with Cu-K_α_ radiation. TLC analysis was performed on pre-coated, glass-backed silica gel plates (Qingdao Haiyang, Qingdao, China) and visualized with UV light. Flash column chromatography was performed on silica gel (200–300 mesh), with petroleum ether and ethyl acetate (Guanghua Corporation, Guangzhou, China) as eluents.

### 3.2. General Methods for the Preparation of 3-Methylenetetrahydrofuran Derivatives

A mixture of Pd(TFA)_2_ (10 mol %), CuCl_2_ (4 equiv.), DIPEA (80 mol%), and HOAc (2 mL) was added to a tube equipped with a stir bar. Then, propargyl alcohols (**1**, 0.2 mmol) and alkenes (**2**, 0.3 mmol) were added to the tube under air and stirred at room temperature for 24 h. After the reaction was finished, the reaction was quenched by saturated aqueous NH_4_Cl and extracted with EtOAc three times. The combined organic layers were dried over anhydrous Na_2_SO_4_ and evaporated under a vacuum. The residue was purified by flash column chromatography on silica gel (eluting with petroleum ether/ethyl acetate) to afford the desired products.

(*Z*)-3-(chloro(phenyl)methylene)-2,2-dimethyl-5-((*E*)-styryl)tetrahydrofuran (**3a**): Alkynol **1a** (0.20 mmol) with buta-1,3-dien-1-ylbenzene **2a** (0.30 mmol, 1.5 equiv.) gave compound **3a** (82%, 53.2 mg) as a yellow oil. ^1^H NMR (400 MHz, CDCl_3_) δ 7.47–7.43 (m, 2H), 7.42–7.38 (m, 4H), 7.37–7.31 (m, 3H), 7.30–7.26 (m, 1H), 6.66 (d, *J* = 15.9 Hz, 1H), 6.25 (dd, *J* = 15.9, 7.0 Hz, 1H), 4.59–4.47 (m, 1H), 2.79–2.64 (m, 2H), 1.76 (s, 3H), 1.71 (s, 3H); ^13^C NMR (100 MHz, CDCl_3_) δ 144.6, 140.0, 136.5, 132.4, 128.8, 128.5, 128.3, 128.3, 128.2, 127.8, 126.6, 121.3, 83.6, 42.1, 26.1, 24.0; HRMS (ESI, *m*/*z*): calcd for C_21_H_21_ClO, [M+H]^+^: 325.1184, found: 325.1179.

(*Z*)-3-(chloro(*p*-tolyl)methylene)-2,2-dimethyl-5-((*E*)-styryl)tetrahydrofuran (**3b**): Alkynol **1b** (0.20 mmol) with buta-1,3-dien-1-ylbenzene **2a** (0.30 mmol, 1.5 equiv.) gave compound **3b** (80%, 54.4 mg) as a yellow oil. ^1^H NMR (400 MHz, CDCl_3_) δ 7.42–7.37 (m, 2H), 7.37–7.31 (m, 2H), 7.27 (td, *J* = 10.7, 8.7, 2.4 Hz, 4H), 7.15 (d, *J* = 7.5 Hz, 1H), 6.65 (d, *J* = 15.9 Hz, 1H), 6.24 (dd, *J* = 15.9, 7.0 Hz, 1H), 4.57–4.49 (m, 1H), 2.71 (d, *J* = 9.3 Hz, 2H), 2.40 (s, 3H), 1.74 (s, 3H), 1.69 (s, 3H); ^13^C NMR (100 MHz, CDCl_3_) δ 144.3, 139.9, 138.0, 136.5, 132.4, 129.0, 128.9, 128.8, 128.5, 128.2, 127.8, 126.6, 125.4, 121.4, 83.6, 76.4, 42.1, 26.1, 24.0, 21.4; HRMS (ESI, *m*/*z*): calcd for C_22_H_23_ClO, [M+H]^+^: 339.1340, found: 339.1345.

(*Z*)-3-((4-(*tert*-butyl)phenyl)chloromethylene)-2,2-dimethyl-5-((*E*)-styryl)tetrahydrofuran (**3c**): Alkynol **1c** (0.20 mmol) with buta-1,3-dien-1-ylbenzene **2a** (0.30 mmol, 1.5 equiv.) gave compound **3c** (76%, 57.5 mg) as a yellow solid, mp = 117.9–118.6 °C; ^1^H NMR (400 MHz, CDCl_3_) δ 7.45–7.37 (m, 5H), 6.65 (d, *J* = 15.9 Hz, 1H), 6.25 (dd, *J* = 15.9, 7.0 Hz, 1H), 4.58–4.48 (m, 1H), 2.83–2.66 (m, 2H), 1.75 (s, 3H), 1.70 (s, 3H), 1.36 (s, 9H); ^13^C NMR (100 MHz, CDCl_3_) δ 151.3, 144.1, 137.0, 136.5, 132.4, 128.9, 128.5, 128.0, 127.8, 126.6, 125.1, 121.4, 83.7, 76.4, 42.2, 34.7, 31.3, 26.2, 24.1; HRMS (ESI, *m*/*z*): calcd for C_25_H_29_ClO, [M+H]^+^: 381.1245, found: 381.1246.

(*Z*)-3-(chloro(4-chlorophenyl)methylene)-2,2-dimethyl-5-((*E*)-styryl)-tetrahydrofuran (**3d**): Alkynol **1d** (0.20 mmol) with buta-1,3-dien-1-ylbenzene **2a** (0.30 mmol, 1.5 equiv.) gave compound **3d** (66%, 47.0 mg) as a yellow oil. ^1^H NMR (400 MHz, CDCl_3_) δ 7.37 (d, *J* = 6.2 Hz, 7H), 7.32–7.27 (m, 2H), 6.64 (d, *J* = 15.9 Hz, 1H), 6.22 (dd, *J* = 15.9, 7.0 Hz, 1H), 4.56–4.49 (m, 1H), 2.68 (d, *J* = 4.5 Hz, 2H), 1.72 (s, 3H), 1.67 (s, 3H); ^13^C NMR (100 MHz, CDCl_3_) δ 145.4, 138.3, 136.4, 134.1, 132.6, 129.7, 128.6, 128.5, 127.9, 126.6, 120.1, 83.7, 76.4, 42.1, 26.0, 23.9; HRMS (ESI, *m*/*z*): calcd for C_21_H_20_Cl_2_O, [M+H]^+^: 359.0794, found: 359.0789.

(*Z*)-3-((4-bromophenyl)chloromethylene)-2,2-dimethyl-5-((*E*)-styryl)-tetrahydrofuran (**3e**): Alkynol **1e** (0.20 mmol) with buta-1,3-dien-1-ylbenzene **2a** (0.30 mmol, 1.5 equiv.) gave compound **3e** (68%, 55.0 mg) as a yellow oil. ^1^H NMR (400 MHz, CDCl_3_) δ 7.54–7.49 (m, 2H), 7.41–7.37 (m, 2H), 7.36–7.26 (m, 5H), 6.64 (d, *J* = 15.9 Hz, 1H), 6.23 (dd, *J* = 15.9, 7.0 Hz, 1H), 4.56–4.47 (m, 1H), 2.73–2.63 (m, 2H), 1.72 (s, 3H), 1.67 (s, 3H); ^13^C NMR (100 MHz, CDCl_3_) δ 145.4, 138.8, 136.4, 132.5, 131.5, 130.0, 128.5, 128.5, 127.9, 126.6, 122.3, 120.2, 83.7, 76.4, 42.1, 26.0, 23.9; HRMS (ESI, *m*/*z*): calcd for C_21_H_20_BrClO, [M+H]^+^: 403.0296, found: 403.0293.

4-((*Z*)-chloro-2,2-dimethyl-5-((*E*)-styryl)dihydrofuran-3(2*H*)-ylidene)methyl)-phenyl acetaten (**3f**): Alkynol **1f** (0.20 mmol) with buta-1,3-dien-1-ylbenzene **2a** (0.30 mmol, 1.5 equiv.) gave compound **3f** (62%, 47.9 mg) as a yellow oil. ^1^H NMR (400 MHz, CDCl_3_) δ 7.46–7.25 (m, 7H), 7.16–7.06 (m, 2H), 6.64 (d, *J* = 15.9 Hz, 1H), 6.23 (dd, *J* = 15.9, 7.0 Hz, 1H), 4.57–4.47 (m, 1H), 2.79–2.62 (m, 2H), 2.33 (s, 3H), 1.72 (s, 3H), 1.67 (s, 3H); ^13^C NMR (100 MHz, CDCl_3_) δ 169.3, 150.3, 145.0, 137.5, 136.4, 132.5, 129.6, 128.6, 128.5, 127.8, 126.6, 121.4, 120.5, 83.7, 76.4, 42.1, 26.1, 23.9, 21.2; HRMS (ESI, *m*/*z*): calcd for C_23_H_23_ClO_3_, [M+H]^+^: 383.1411, found: 383.1408.

methyl 4-((*Z*)-chloro(2,2-dimethyl-5-((*E*)-styryl)dihydrofuran-3(2*H*)-ylidene) methyl)benzoate (**3g**): Alkynol **1g** (0.20 mmol) with buta-1,3-dien-1-ylbenzene **2a** (0.30 mmol, 1.5 equiv.) gave compound **3g** (60%, 45.8 mg) as a yellow oil. ^1^H NMR (400 MHz, CDCl_3_) δ 8.06 (d, *J* = 8.2 Hz, 2H), 7.51 (d, *J* = 8.1 Hz, 2H), 7.38 (d, *J* = 6.9 Hz, 2H), 7.32 (t, *J* = 7.7 Hz, 3H), 6.64 (d, *J* = 15.9 Hz, 1H), 6.23 (dd, *J* = 15.9, 7.0 Hz, 1H), 4.57–4.49 (m, 1H), 3.95 (s, 3H), 2.71 (d, *J* = 7.7 Hz, 2H), 1.74 (s, 3H); ^13^C NMR (100 MHz, CDCl_3_) δ 166.5, 146.1, 144.2, 132.6, 129.8, 129.6, 128.5, 128.4, 127.9, 126.6, 120.2, 83.8, 76.4, 52.3, 42.1, 26.1, 23.9; HRMS (ESI, *m*/*z*): calcd for C_23_H_23_ClO_3_, [M+H]^+^: 383.1410, found: 383.1408.

4-((*Z*)-Chloro(2,2-dimethyl-5-((*E*)-styryl)dihydrofuran-3(2*H*)-ylidene)methyl) benzaldehyde (**3h**): Alkynol **1h** (0.20 mmol) with buta-1,3-dien-1-ylbenzene **2a** (0.30 mmol, 1.5 equiv.) gave compound **3h** (58%, 41.1 mg) as a yellow oil. ^1^H NMR (400 MHz, CDCl_3_) δ 10.04 (s, 1H), 7.95–7.87 (m, 2H), 7.64–7.58 (m, 2H), 7.41–7.36 (m, 2H), 7.34–7.28 (m, 2H), 7.28–7.20 (m, 1H), 6.64 (d, *J* = 15.9 Hz, 1H), 6.23 (dd, *J* = 15.9, 7.0 Hz, 1H), 4.57–4.50 (m, 1H), 2.72 (d, *J* = 7.6 Hz, 2H), 1.74 (s, 3H), 1.68 (s, 3H); ^13^C NMR (100 MHz, CDCl_3_) δ 191.5, 146.7, 145.6, 136.3, 135.8, 132.7, 129.7, 129.1, 128.6, 128.3, 127.9, 126.6, 120.0, 83.9, 42.1, 26.1, 23.8; HRMS (ESI, *m*/*z*): calcd for C_22_H_21_ClO_2_, [M+H]^+^: 353.1306, found: 353.1303.

(*Z*)-3-(chloro(4-(trifluoromethoxy)phenyl)methylene)-2,2-dimethyl-5-((*E*)-styryl) tetrahydrofuran (**3i**): Alkynol **1i** (0.20 mmol) with buta-1,3-dien-1-ylbenzene **2a** (0.30 mmol, 1.5 equiv.) gave compound **3i** (56%, 45.5 mg) as a yellow oil. ^1^H NMR (400 MHz, CDCl_3_) δ 7.46 (d, *J* = 8.5 Hz, 2H), 7.39 (d, *J* = 7.5 Hz, 2H), 7.33 (d, *J* = 7.4 Hz, 2H), 7.29–7.22 (m, 3H), 6.65 (d, *J* = 15.9 Hz, 1H), 6.23 (dd, *J* = 15.9, 7.1 Hz, 1H), 4.57–4.48 (m, 1H), 2.74–2.65 (m, 2H), 1.73 (s, 3H); ^13^C NMR (100 MHz, CDCl_3_) δ 148.7, 145.6, 138.5, 136.4, 132.6, 129.9, 128.5, 128.5, 127.9, 126.6, 120.8, 119.9, 83.7, 42.1, 26.0, 23.9; ^19^F NMR (376 MHz, CDCl_3_) δ −57.80; HRMS (ESI, *m*/*z*): calcd for C_22_H_20_ClF_3_O_2_, [M+H]^+^: 409.1000, found: 409.1002.

(*Z*)-3-(chloro(4-(trifluoromethyl)phenyl)methylene)-2,2-dimethyl-5-((*E*)-styryl) tetrahydrofuran (**3j**): Alkynol **1j** (0.20 mmol) with buta-1,3-dien-1-ylbenzene **2a** (0.30 mmol, 1.5 equiv.) gave compound **3j** (55%, 42.8 mg) as a yellow oil. ^1^H NMR (400 MHz, CDCl_3_) δ 7.65 (d, *J* = 8.2 Hz, 2H), 7.55 (d, *J* = 8.1 Hz, 2H), 7.38 (d, *J* = 7.7 Hz, 2H), 7.32 (t, *J* = 7.5 Hz, 2H), 7.29–7.25 (m, 1H), 6.64 (d, *J* = 15.9 Hz, 1H), 6.22 (dd, *J* = 15.9, 7.0 Hz, 1H), 4.58–4.49 (m, 1H), 2.70 (d, *J* = 7.6 Hz, 2H), 1.74 (s, 3H), 1.68 (s, 3H); ^13^C NMR (100 MHz, CDCl_3_) δ 146.3, 143.3, 136.3, 132.7, 128.8, 128.5, 128.4, 127.9, 126.6, 125.3 (dd, *J* = 8.3, 4.0 Hz), 83.8, 42.1, 26.0, 23.8; ^19^F NMR (376 MHz, CDCl_3_) δ −62.75; HRMS (ESI, *m*/*z*): calcd for C_22_H_20_ClF_3_O, [M+H]^+^: 393.1053, found: 393.1054.

(*Z*)-3-(chloro(3-methoxyphenyl)methylene)-2,2-dimethyl-5-((*E*)-styryl) tetrahydrofuran (**3k**): Alkynol **1k** (0.20 mmol) with buta-1,3-dien-1-ylbenzene **2a** (0.30 mmol, 1.5 equiv.) gave compound **3k** (69%, 48.0 mg) as a yellow oil. ^1^H NMR (400 MHz, CDCl_3_) *δ* 7.39 (dt, *J* = 6.6, 1.4 Hz, 2H), 7.36–7.25 (m, 4H), 7.06–6.93 (m, 2H), 6.88 (ddd, *J* = 8.3, 2.7, 1.1 Hz, 1H), 6.64 (d, *J* = 15.9 Hz, 1H), 6.23 (dd, *J* = 15.9, 7.0 Hz, 1H), 4.57–4.48 (m, 1H), 3.85 (s, 3H), 2.80–2.62 (m, 2H), 1.73 (s, 3H), 1.68 (s, 3H); ^13^C NMR (100 MHz, CDCl_3_) δ 159.4, 144.7, 141.2, 136.5, 132.4, 129.3, 128.7, 128.5, 127.8, 126.6, 121.1, 120.7, 114.0, 113.9, 83.6, 76.4, 55.3, 42.1, 26.1, 23.9; HRMS (ESI, *m*/*z*): calcd for C_22_H_23_ClO_2_, [M+H]^+^: 355.1291, found: 355.1284.

(*Z*)-3-((3-bromophenyl)chloromethylene)-2,2-dimethyl-5-((*E*)-styryl) tetrahydrofuran (**3l**): Alkynol **1l** (0.20 mmol) with buta-1,3-dien-1-ylbenzene **2a** (0.30 mmol, 1.5 equiv.) gave compound **3l** (65%, 52.0 mg) as a yellow oil. ^1^H NMR (400 MHz, CDCl_3_) δ 7.59 (t, *J* = 1.9 Hz, 1H), 7.47 (dt, *J* = 8.1, 1.5 Hz, 1H), 7.41–7.30 (m, 5H), 7.26 (t, *J* = 7.7 Hz, 2H), 6.65 (d, *J* = 15.9 Hz, 1H), 6.23 (dd, *J* = 15.9, 7.1 Hz, 1H), 4.58–4.48 (m, 1H), 2.76–2.62 (m, 2H), 1.72 (s, 3H), 1.67 (s, 3H); ^13^C NMR (100 MHz, CDCl_3_) δ 145.9, 141.8, 136.4, 132.6, 131.4, 131.3, 129.9, 128.6, 128.5, 127.9, 127.0, 126.6, 122.3, 119.7, 83.7, 77.4, 77.1, 76.7, 76.4, 42.1, 26.0, 23.9; HRMS (ESI, *m*/*z*): calcd for C_21_H_20_BrClO, [M+H]^+^: 403.0297, found: 403.0293.

(*Z*)-3-(chloro(3-chlorophenyl)methylene)-2,2-dimethyl-5-((*E*)-styryl) tetrahydrofuran (**3m**): Alkynol **1m** (0.20 mmol) with buta-1,3-dien-1-ylbenzene **2a** (0.30 mmol, 1.5 equiv.) gave compound **3m** (60%, 43.2 mg) as a yellow oil. ^1^H NMR (400 MHz, CDCl_3_) δ 7.45–7.37 (m, 3H), 7.36–7.25 (m, 6H), 6.66 (d, *J* = 15.9 Hz, 1H), 6.24 (dd, *J* = 15.9, 7.1 Hz, 1H), 4.57–4.48 (m, 1H), 2.77–2.65 (m, 2H), 1.74 (s, 3H), 1.68 (s, 3H); ^13^C NMR (100 MHz, CDCl_3_) δ 145.8, 141.5, 136.4, 134.2, 132.6, 129.6, 128.6, 128.5, 128.4, 127.8, 126.6, 126.6, 120.2, 83.7, 76.4, 42.1, 26.1, 23.9; HRMS (ESI, *m*/*z*): calcd for C_21_H_20_Cl_2_O, [M+H]^+^: 359.0794, found: 359.0789.

(*Z*)-3-(chloro(3-nitrophenyl)methylene)-2,2-dimethyl-5-((*E*)-styryl) tetrahydrofuran (**3n**): Alkynol **1n** (0.20 mmol) with buta-1,3-dien-1-ylbenzene **2a** (0.30 mmol, 1.5 equiv.) gave compound **3n** (53%, 39.2 mg) as a yellow oil. ^1^H NMR (400 MHz, CDCl_3_) δ 8.31 (t, *J* = 2.0 Hz, 1H), 8.22–8.14 (m, 1H), 7.80–7.74 (m, 1H), 7.58 (t, *J* = 8.0 Hz, 1H), 7.40–7.36 (m, 2H), 7.35–7.26 (m, 3H), 6.65 (d, *J* = 15.9 Hz, 1H), 6.23 (dd, *J* = 15.9, 7.1 Hz, 1H), 4.56–4.46 (m, 1H), 2.78–2.67 (m, 2H), 1.74 (s, 3H), 1.68 (s, 3H); ^13^C NMR (100 MHz, CDCl_3_) δ 148.1, 147.3, 141.4, 136.3, 134.4, 132.9, 129.4, 128.6, 128.2, 127.9, 126.6, 123.5, 123.1, 118.7, 83.8, 76.4, 42.0, 25.9, 23.8; HRMS (ESI, *m*/*z*): calcd for C_21_H_20_ClNO_3_, [M+H]^+^: 370.1036, found: 370.1029.

(*Z*)-3-(chloro(2-chlorophenyl)methylene)-2,2-dimethyl-5-((*E*)-styryl) tetrahydrofuran (**3o**): Alkynol **1o** (0.20 mmol) with buta-1,3-dien-1-ylbenzene **2a** (0.30 mmol, 1.5 equiv.) gave compound **3o** (50%, 35.6 mg) as a yellow oil. ^1^H NMR (400 MHz, CDCl_3_) δ 7.48–7.43 (m, 1H), 7.36 (d, *J* = 7.2 Hz, 2H), 7.33–7.28 (m, 5H), 7.25 (d, *J* = 7.0 Hz, 1H), 6.62 (dd, *J* = 15.9, 5.6 Hz, 1H), 6.26–6.17 (m, 1H), 4.64–4.52 (m, 1H), 2.54–2.33 (m, 2H), 1.75 (s, 3H), 1.69 (s, 3H); ^13^C NMR (100 MHz, CDCl_3_) δ 138.5, 136.5, 132.4, 130.0, 128.7, 128.5, 127.8, 127.3, 126.5; HRMS (ESI, *m*/*z*): calcd for C_21_H_20_Cl_2_O, [M+H]^+^: 359.0792, found: 359.0789.

(*Z*)-3-(chloro(3,5-dimethylphenyl)methylene)-2,2-dimethyl-5-((*E*)-styryl) tetrahydrofuran (**3p**): Alkynol **1p** (0.20 mmol) with buta-1,3-dien-1-ylbenzene **2a** (0.30 mmol, 1.5 equiv.) gave compound **3p** (77%, 54.0 mg) as a yellow oil. ^1^H NMR (400 MHz, CDCl_3_) δ 7.40 (d, *J* = 7.3 Hz, 2H), 7.33 (t, *J* = 7.5 Hz, 2H), 7.29–7.24 (m, 1H), 7.05 (s, 2H), 6.98 (s, 1H), 6.66 (d, *J* = 15.9 Hz, 1H), 6.26 (dd, *J* = 15.9, 7.0 Hz, 1H), 4.59–4.49 (m, 1H), 2.80–2.66 (m, 2H), 2.37 (s, 6H), 1.75 (s, 3H); ^13^C NMR (100 MHz, CDCl_3_) δ 144.1, 139.9, 137.9, 136.5, 132.3, 129.9, 128.9, 128.5, 127.8, 126.6, 126.0, 121.6, 83.6, 76.4, 42.1, 26.2, 24.1, 21.3; HRMS (ESI, *m*/*z*): calcd for C_23_H_25_ClO, [M+H]^+^: 353.1666, found: 353.1667.

(*Z*)-3-((4-bromo-2-fluorophenyl)chloromethylene)-2,2-dimethyl-5-((*E*)-styryl) tetrahydrofuran (**3q**): Alkynol **1q** (0.20 mmol) with buta-1,3-dien-1-ylbenzene **2a** (0.30 mmol, 1.5 equiv.) gave compound **3q** (61%, 50.6 mg) as a yellow oil. ^1^H NMR (400 MHz, CDCl_3_) δ 7.39–7.36 (m, 2H), 7.35–7.31 (m, 3H), 7.31–7.24 (m, 3H), 6.64 (d, *J* = 15.9 Hz, 1H), 6.26–6.13 (m, 1H), 4.60–4.52 (m, 1H), 2.59–2.48 (m, 2H), 1.72 (s, 3H), 1.66 (s, 3H); ^13^C NMR (100 MHz, CDCl_3_) δ 158.7 (d, *J* = 254.2 Hz), 148.6, 136.4, 132.6, 131.7 (d, *J* = 3.3 Hz), 128.5 (d, *J* = 3.5 Hz), 127.9, 127.8 (d, *J* = 3.7 Hz), 126.6, 126.5, 123.0 (d, *J* = 9.3 Hz), 119.8 (d, *J* = 25.1 Hz), 113.5, 83.5, 76.3, 41.3, 41.2, 26.1, 23.8; ^19^F NMR (376 MHz, CDCl_3_) δ -110.71; HRMS (ESI, *m*/*z*): calcd for C_21_H_19_BrClFO, [M+H]^+^: 421.0363, found: 421.0365.

(*Z*)-3-(chloro(naphthalen-1-yl)methylene)-2,2-dimethyl-5-((*E*)-styryl) tetrahydrofuran (**3r**): Alkynol **1r** (0.20 mmol) with buta-1,3-dien-1-ylbenzene **2a** (0.30 mmol, 1.5 equiv.) gave compound **3r** (40%, 54.0 mg) as a yellow oil. ^1^H NMR (400 MHz, CDCl_3_) δ 8.0 (d, *J* = 8.2 Hz, 1H), 7.9 (d, *J* = 8.2 Hz, 4H), 7.6–7.4 (m, 3H), 7.3–7.2 (m, 4H), 6.6 (d, *J* = 16.0 Hz, 1H), 6.25–6.12 (m, 1H), 4.61–4.53 (m, 1H), 2.60–2.49 (m, 2H), 1.90 (s, 3H), 1.78 (s, 3H); ^13^C NMR (100 MHz, CDCl_3_) δ 146.9, 137.4, 136.4, 134.0, 132.3, 130.0, 129.0, 128.7, 128.6, 128.5, 127.8, 127.0, 126.8, 126.4, 126.3, 125.6, 124.6, 119.2, 83.4, 76.4, 41.5, 41.1, 26.4, 24.0; HRMS (ESI, *m*/*z*): calcd for C_25_H_23_ClO, [M+H]^+^: 375.1340, found: 375.1335.

(*Z*)-3-(chloro(thiophen-2-yl)methylene)-2,2-dimethyl-5-((*E*)-styryl) tetrahydrofuran (**3s**): Alkynol **1s** (0.20 mmol) with buta-1,3-dien-1-ylbenzene **2a** (0.30 mmol, 1.5 equiv.) gave compound **3s** (77%, 51.0 mg) as a yellow oil. ^1^H NMR (400 MHz, CDCl_3_) δ 7.46–7.41 (m, 2H), 7.41–7.31 (m, 3H), 7.31–7.21 (m, 2H), 7.06 (dd, *J* = 5.1, 3.8 Hz, 1H), 6.72 (d, *J* = 15.9 Hz, 1H), 6.31 (dd, *J* = 15.9, 7.1 Hz, 1H), 4.67–4.59 (m, 1H), 3.13 (dd, *J* = 16.2, 5.4 Hz, 1H), 2.82 (dd, *J* = 16.2, 5.4 Hz, 1H), 1.74 (s, 3H), 1.67 (s, 3H); ^13^C NMR (100 MHz, CDCl_3_) δ 144.6, 142.2, 136.5, 132.6, 128.7, 128.6, 127.9, 127.4, 126.76, 126.6, 126.2, 116.1, 84.5, 76.4, 42.7, 26.2, 23.9; HRMS (ESI, *m*/*z*): calcd for C_19_H_19_ClOS, [M+H]^+^: 331.0747, found: 331.0743.

(*Z*)-3-(chloro(cyclopropyl)methylene)-2,2-dimethyl-5-((*E*)-styryl) tetrahydrofuran (**3t**): Alkynol **1t** (0.20 mmol) with buta-1,3-dien-1-ylbenzene **2a** (0.30 mmol, 1.5 equiv.) gave compound **3t** (59%, 34.0 mg) as a yellow oil. ^1^H NMR (400 MHz, CDCl_3_) δ 7.43 (d, *J* = 7.2 Hz, 2H), 7.34 (t, *J* = 7.4 Hz, 2H), 7.27 (t, *J* = 7.2 Hz, 1H), 6.70 (d, *J* = 15.9 Hz, 1H), 6.29 (dd, *J* = 15.9, 7.0 Hz, 1H), 4.64–4.56 (m, 1H), 3.01 (dd, *J* = 15.6, 5.4 Hz, 1H), 2.65 (dd, *J* = 15.5, 5.4 Hz, 1H), 1.75 -1.69 (m, 1H), 1.61 (s, 3H), 1.54 (s, 3H), 0.92–0.81 (m, 2H), 0.78–0.70 (m, 1H); ^13^C NMR (100 MHz, CDCl_3_) δ 141.1, 136.6, 132.3, 129.2, 128.5, 127.8, 126.6, 124.4, 83.5, 76.19, 40.2, 26.4, 24.0, 16.6, 5.6, 5.4; HRMS (ESI, *m*/*z*): calcd for C_18_H_21_ClO, [M+H]^+^: 289.1355, found: 289.1354.

(*Z*)-3-(1-chlorobutylidene)-2,2-dimethyl-5-((*E*)-styryl)tetrahydrofuran (**3u**): Alkynol **1u** (0.20 mmol) with buta-1,3-dien-1-ylbenzene **2a** (0.30 mmol, 1.5 equiv.) gave compound **3u** (75%, 43.1 mg) as a yellow oil. ^1^H NMR (400 MHz, CDCl_3_) δ 7.46–7.39 (m, 2H), 7.37–7.31 (m, 2H), 7.29–7.24 (m, 1H), 6.69 (d, *J* = 15.9 Hz, 1H), 6.26 (dd, *J* = 15.9, 7.1 Hz, 1H), 4.61–4.51 (m, 1H), 2.86 (dd, *J* = 15.4, 5.5 Hz, 1H), 2.55 (dd, *J* = 15.5, 10.2 Hz, 1H), 2.34 (t, *J* = 7.2 Hz, 2H), 1.74–1.64 (m, 2H), 1.63 (s, 3H), 1.52 (s, 3H), 0.96 (t, *J* = 7.4 Hz, 3H); ^13^C NMR (100 MHz, CDCl_3_) δ 141.6, 136.5, 132.3, 129.1, 128.5, 127.8, 126.6, 124.1, 83.2, 76.0, 40.4, 39.9, 26.4, 23.9, 20.4, 13.0; HRMS (ESI, *m*/*z*): calcd for C_18_H_23_ClO, [M+H]^+^: 291.1511, found: 291.1510.

(*Z*)-3-(1-chlorohexylidene)-2,2-dimethyl-5-((*E*)-styryl)tetrahydrofuran (**3v**): Alkynol **1v** (0.20 mmol) with buta-1,3-dien-1-ylbenzene **2a** (0.30 mmol, 1.5 equiv.) gave compound **3v** (80%, 50.9 mg) as a yellow oil. ^1^H NMR (400 MHz, CDCl_3_) δ 7.43–7.39 (m, 2H), 7.36–7.30 (m, 2H), 7.29–7.25 (m, 1H), 6.67 (d, *J* = 15.9 Hz, 1H), 6.25 (dd, *J* = 15.9, 7.1 Hz, 1H), 4.62–4.52 (m, 1H), 3.02 (dd, *J* = 16.6, 5.7 Hz, 1H), 2.74–2.57 (m, 1H), 2.44–2.31 (m, 2H), 1.70–1.57 (m, 3H), 1.53 (s, 3H), 1.45 (s, 3H), 1.42–1.28 (m, 5H), 0.95 (t, *J* = 6.9 Hz, 3H); ^13^C NMR (100 MHz, CDCl_3_) δ 142.6, 136.6, 132.2, 129.2, 128.5, 127.7, 127.4, 126.6, 81.8, 75.7, 42.0, 35.3, 31.3, 28.2, 27.9, 26.2, 22.5, 14.0, 14.0; HRMS (ESI, *m*/*z*): calcd for C_20_H_27_ClO, [M+H]^+^: 319.1826, found: 319.1823.

(*Z*)-3-(1-chlorononylidene)-2,2-dimethyl-5-((*E*)-styryl)tetrahydrofuran (**3w**): Alkynol **1w** (0.20 mmol) with buta-1,3-dien-1-ylbenzene **2a** (0.30 mmol, 1.5 equiv.) gave compound **3w** (77%, 55.0 mg) as a yellow solid, mp = 69.4–70.2 °C; ^1^H NMR (400 MHz, CDCl_3_) δ 7.42 (d, *J* = 7.1 Hz, 2H), 7.33 (t, *J* = 7.5 Hz, 2H), 7.29–7.25 (m, 1H), 6.67 (d, *J* = 15.9 Hz, 1H), 6.25 (dd, *J* = 15.9, 7.1 Hz, 1H), 4.63–4.53 (m, 1H), 3.02 (dd, *J* = 16.6, 5.6 Hz, 1H), 2.69–2.57 (m, 1H), 2.46–2.31 (m, 2H), 1.65–1.58 (m, 2H), 1.45 (s, 3H), 1.39–1.29 (m, 10H), 0.92 (t, *J* = 6.6 Hz, 3H); ^13^C NMR (100 MHz, CDCl_3_) δ 142.6, 136.6, 132.2, 129.2, 128.5, 127.7, 127.4, 126.6, 81.8, 75.7, 42.0, 35.3, 31.9, 29.5, 29.2, 29.2, 28.3, 28.2, 26.2, 22.7, 14.1; HRMS (ESI, *m*/*z*): calcd for C_23_H_33_ClO, [M+H]^+^: 361.2294, found: 361.2293.

(*Z*)-4-(chloro(phenyl)methylene)-2-((*E*)-styryl)tetrahydrofuran (**3x**): Alkynol **1x** (0.20 mmol) with buta-1,3-dien-1-ylbenzene **2a** (0.30 mmol, 1.5 equiv.) gave compound **3x** (57%, 34.0 mg) as a yellow oil. ^1^H NMR (400 MHz, CDCl_3_) δ 7.55–7.48 (m, 2H), 7.41 (dd, *J* = 7.9, 2.8 Hz, 4H), 7.34 (t, *J* = 7.5 Hz, 3H), 7.30–7.26 (m, 1H), 6.66 (d, *J* = 15.9 Hz, 1H), 6.27 (dd, *J* = 15.9, 6.7 Hz, 1H), 4.84–4.74 (m, 1H), 4.70–4.54 (m, 2H), 3.00–2.85 (m, 1H), 2.76–2.62 (m, 1H); ^13^C NMR (100 MHz, CDCl_3_) δ 138.1, 137.9, 136.4, 132.2, 128.6, 128.4, 128.3, 128.2, 127.9, 127.9, 126.6, 121.9, 81.4, 72.1, 39.5; HRMS (ESI, *m*/*z*): calcd for C_19_H_17_ClO, [M+H]^+^: 297.1043, found: 297.1041.

(*Z*)-3-(chloro(phenyl)methylene)-2-methyl-5-((*E*)-styryl)tetrahydrofuran (**3y**): Alkynol **1y** (0.20 mmol) with buta-1,3-dien-1-ylbenzene **2a** (0.30 mmol, 1.5 equiv.) gave compound **3y** (53%, 33.0 mg) as a yellow oil. ^1^H NMR (400 MHz, CDCl_3_) δ 7.42 (t, *J* = 7.3 Hz, 4H), 7.34 (dd, *J* = 8.8, 6.1 Hz, 5H), 7.28 (d, *J* = 5.5 Hz, 1H), 6.71 (d, *J* = 15.9 Hz, 1H), 6.32 (dd, *J* = 15.9, 6.1 Hz, 1H), 4.59–4.52 (m, 1H), 4.50–4.44 (m, 1H), 2.77–2.67 (m, 1H), 2.57–2.49 (m, 1H), 1.59 (d, *J* = 6.5 Hz, 3H); ^13^C NMR (100 MHz, CDCl_3_) δ 139.4, 136.5, 132.3, 131.5, 129.8, 128.6, 128.5, 128.3, 128.1, 127.8, 127.6, 126.6, 38.8, 20.3; HRMS (ESI, *m*/*z*): calcd for C_20_H_19_ClO, [M+H]^+^: 311.1199, found: 311.1197.

(*Z*)-3-(chloro(phenyl)methylene)-2-phenyl-5-((*E*)-styryl)tetrahydrofuran (**3aa**): Alkynol **1aa** (0.20 mmol) with buta-1,3-dien-1-ylbenzene **2a** (0.30 mmol, 1.5 equiv.) gave compound **3aa** (47%, 35.0 mg) as a yellow oil. ^1^H NMR (400 MHz, CDCl_3_) δ 7.65–7.53 (m, 3H), 7.53–7.43 (m, 8H), 7.42–7.31 (m, 4H), 7.30 (dd, *J* = 2.9, 1.5 Hz, 1H), 6.74 (d, *J* = 16.0 Hz, 1H), 6.38 (dd, *J* = 16.0, 6.2 Hz, 1H), 5.50 (s, 1H), 4.56–4.49 (m, 1H), 2.99–2.91 (m, 1H), 2.76–2.73 (m, 1H); ^13^C NMR (100 MHz, CDCl_3_) δ 139.4, 139.1, 139.0, 138.2, 136.5, 134.3, 133.8, 131.7, 131.5, 129.3, 128.8, 128.7, 128.6, 128.5, 128.4, 128.4, 128.2, 128.1, 128.1, 127.9, 127.9, 127.8, 126.6, 126.5, 125.8, 81.5, 78.7, 68.1, 38.8, 38.2; HRMS (ESI, *m*/*z*): calcd for C_25_H_21_ClO, [M+H]^+^: 373.1356, found: 373.1354.

(*Z*)-4-(chloro(phenyl)methylene)-2-((*E*)-styryl)-1-oxaspiro[4.4]nonane (**3ab**): Alkynol **1ab** (0.20 mmol) with buta-1,3-dien-1-ylbenzene **2a** (0.30 mmol, 1.5 equiv.) gave compound **3ab** (55%, 39.0 mg) as a yellow oil. ^1^H NMR (400 MHz, CDCl_3_) δ 7.48–7.42 (m, 2H), 7.41–7.36 (m, 4H), 7.35–7.29 (m, 3H), 7.29–7.24 (m, 1H), 6.63 (d, *J* = 15.9 Hz, 1H), 6.26 (dd, *J* = 15.9, 7.1 Hz, 1H), 4.43–4.35 (m, 1H), 2.72–2.68 (m, 2H), 2.44–2.34 (m, 1H), 2.06–1.80 (m, 7H); ^13^C NMR (100 MHz, CDCl_3_) δ 143.7, 140.0, 136.5, 132.4, 128.7, 128.5, 128.3, 128.2, 128.2, 127.8, 126.6, 121.1, 93.9, 76.9, 42.4, 37.6, 36.5, 25.9, 25.5; HRMS (ESI, *m*/*z*): calcd for C_23_H_23_ClO, [M+H]^+^: 351.1336, found: 351.1335.

(*Z*)-4-(chloro(phenyl)methylene)-2-((*E*)-styryl)-1-oxaspiro[4.5]decane (**3ac**): Alkynol **1ac** (0.20 mmol) with buta-1,3-dien-1-ylbenzene **2a** (0.30 mmol, 1.5 equiv.) gave compound **3ac** (50%, 36.0 mg) as a yellow oil. ^1^H NMR (400 MHz, CDCl_3_) δ 7.44–7.35 (m, 6H), 7.34–7.28 (m, 3H), 7.25 (d, *J* = 7.2 Hz, 1H), 6.61 (d, *J* = 15.9 Hz, 1H), 6.25 (dd, *J* = 15.9, 6.8 Hz, 1H), 4.50–4.39 (m, 1H), 2.71–2.59 (m, 2H), 2.57–2.47 (m, 1H), 2.39–2.29 (m, 1H), 1.82–1.62 (m, 7H), 1.36–1.30 (m, 1H); ^13^C NMR (100 MHz, CDCl_3_) δ 144.5, 140.5, 136.6, 131.8, 129.3, 128.5, 128.4, 128.3, 128.1, 127.7, 126.5, 121.1, 85.1, 76.1, 42.3, 33.5, 29.8, 25.2, 22.3, 22.1; HRMS (ESI, *m*/*z*): calcd for C_24_H_25_ClO, [M+H]^+^: 365.1492, found: 365.1494.

(*Z*)-3-(chloro(naphthalen-1-yl)methylene)-2-methyl-5-((*E*)-styryl)tetrahydrofuran (**3ae**): Alkynol **1ae** (0.20 mmol) with buta-1,3-dien-1-ylbenzene **2a** (0.30 mmol, 1.5 equiv.) gave compound **3ae** (45%, 34.0 mg) as a yellow oil. ^1^H NMR (400 MHz, CDCl_3_) δ 7.65 (d, *J* = 7.8 Hz, 4H), 7.53–7.42 (m, 5H), 7.39–7.33 (m, 2H), 7.32–7.27 (m, 1H), 6.65 (d, *J* = 15.9 Hz, 1H), 6.28 (dd, *J* = 15.9, 6.8 Hz, 1H), 4.53–4.42 (m, 1H), 2.82–2.57 (m, 2H), 1.64 (s, 3H); ^13^C NMR (100 MHz, CDCl_3_) δ 140.7, 140.4, 138.3, 138.1, 136.6, 131.9, 131.6, 131.5, 130.9, 130.0, 129.6, 128.8, 128.6, 128.6, 128.5, 128.5, 127.9, 127.4, 127.1, 127.0, 127.0, 126.6, 126.6, 74.3, 72.7, 68.6, 38.7, 38.2, 20.3, 18.4; HRMS (ESI, *m*/*z*): calcd for C_25_H_23_ClO, [M+H]^+^: 375.1319, found: 375.1324.

(*Z*)-4-(chloro(4-chlorophenyl)methylene)-2-((*E*)-styryl)-1-oxaspiro[4.4]nonane (**3af**): Alkynol **1af** (0.20 mmol) with buta-1,3-dien-1-ylbenzene **2a** (0.30 mmol, 1.5 equiv.) gave compound **3af** (44%, 34.0 mg) as a yellow oil. ^1^H NMR (400 MHz, CDCl_3_) δ 7.40 (s, 1H), 7.39–7.35 (m, 5H), 7.34–7.30 (m, 2H), 7.29–7.27 (m, 1H), 6.63 (d, *J* = 15.9 Hz, 1H), 6.25 (dd, *J* = 15.9, 7.0 Hz, 1H), 4.46–4.34 (m, 1H), 2.72–2.66 (m, 2H), 2.64–2.59 (m, 1H), 2.41–2.30 (m, 1H), 2.00–1.83 (m, 6H); ^13^C NMR (100 MHz, CDCl_3_) δ 144.5, 138.4, 136.4, 134.0, 132.5, 129.7, 128.5, 128.5, 127.8, 126.6, 119.9, 93.9, 76.8, 42.4, 37.6, 36.4, 25.9, 25.5; HRMS (ESI, *m*/*z*): calcd for C_23_H_22_Cl_2_O, [M+H]^+^: 385.0945, found: 385.0944.

(*Z*)-4-(chloro(4-chlorophenyl)methylene)-2-((*E*)-styryl)-1-oxaspiro[4.5]decane (**3ag**): Alkynol **1ag** (0.20 mmol) with buta-1,3-dien-1-ylbenzene **2a** (0.30 mmol, 1.5 equiv.) gave compound **3ag** (48%, 38.4 mg) as a yellow oil. ^1^H NMR (400 MHz, CDCl_3_) δ 7.41–7.38 (m, 2H), 7.36–7.33 (m, 4H), 7.31 (d, *J* = 8.0 Hz, 2H), 7.28–7.25 (m, 1H), 6.62 (d, *J* = 15.9 Hz, 1H), 6.25 (dd, *J* = 15.9, 6.9 Hz, 1H), 4.49–4.41 (m, 1H), 2.68–2.58 (m, 2H), 2.54–2.45 (m, 1H), 2.36–2.26 (m, 1H), 1.81- 1.65 (m, 7H), 1.36–1.30 (m, 1H); ^13^C NMR (100 MHz, CDCl_3_) δ 145.3, 138.8, 136.5, 133.9, 131.9, 129.8, 129.1, 128.5, 127.8, 126.6, 119.9, 85.2, 76.1, 42.3, 33.4, 29.7, 25.2, 22.3, 22.1; HRMS (ESI, *m*/*z*): calcd for C_24_H_25_ClO, [M+H]^+^: 399.1106, found: 399.1102.

(*Z*)-4-((4-bromophenyl)chloromethylene)-2-((*E*)-styryl)-1-oxaspiro[4.5]decane (**3ah**): Alkynol **1ah** (0.20 mmol) with buta-1,3-dien-1-ylbenzene **2a** (0.30 mmol, 1.5 equiv.) gave compound **3ah** (45%, 40.0 mg) as a yellow oil. ^1^H NMR (400 MHz, CDCl_3_) δ 7.55–7.47 (m, 2H), 7.41–7.37 (m, 2H), 7.34–7.27 (m, 4H), 6.62 (d, *J* = 15.9 Hz, 1H), 6.25 (dd, *J* = 15.9, 6.8 Hz, 1H), 4.49–2.41 (m, 1H), 2.69–2.55 (m, 2H), 2.53–2.43 (m, 1H), 2.36–2.26 (m, 1H), 1.83–1.63 (m, 7H), 1.36–1.30 (m, 1H); ^13^C NMR (100 MHz, CDCl_3_) δ 145.3, 139.3, 136.5, 131.9, 131.5, 130.1, 129.1, 128.5, 127.8, 126.6, 122.2, 119.9, 85.2, 76.1, 42.3, 33.4, 29.7, 25.2, 22.3, 22.1; HRMS (ESI, *m*/*z*): calcd for C_24_H_24_BrClO, [M+H]^+^: 443.0607, found: 443.0606.

(*Z*)-3-(chloro(phenyl)methylene)-2,2-dimethyl-5-((*E*)-4-methylstyryl) tetrahydrofuran (**4a**): 1-(Buta-1,3-dien-1-yl)-4-methylbenzene **2b** (0.30 mmol, 1.5 equiv.) with alkynol 2-methyl-4-phenylbut-3-yn-2-ol **1a** (0.20 mmol) gave compound **4a** (75%, 51.0 mg) as a yellow oil. ^1^H NMR (400 MHz, CDCl_3_) δ 7.46–7.33 (m, 5H), 7.29 (d, *J* = 8.0 Hz, 2H), 7.13 (d, *J* = 8.1 Hz, 2H), 6.61 (d, *J* = 15.9 Hz, 1H), 6.19 (dd, *J* = 15.9, 7.1 Hz, 1H), 4.55–4.48 (m, 1H), 2.71 (dd, *J* = 7.8, 3.2 Hz, 2H), 2.36 (s, 3H), 1.74 (s, 3H), 1.69 (s, 3H); ^13^C NMR (100 MHz, CDCl_3_) δ 144.7, 139.9, 137.6, 133.7, 132.4, 129.2, 128.3, 128.3, 128.2, 127.7, 126.5, 121.2, 83.6, 76.5, 42.2, 26.1, 24.0, 21.2; HRMS (ESI, *m*/*z*): calcd for C_22_H_23_ClO, [M+H]^+^: 339.0754, found: 339.0755.

(*Z*)-3-(chloro(phenyl)methylene)-2,2-dimethyl-5-((*E*)-3-methylstyryl) tetrahydrofuran (**4b**): 1-(Buta-1,3-dien-1-yl)-3-methylbenzene **2c** (0.30 mmol, 1.5 equiv.) with alkynol 2-methyl-4-phenylbut-3-yn-2-ol **1a** (0.20 mmol) gave compound **4b** (72%, 49.0 mg) as a yellow oil. ^1^H NMR (400 MHz, CDCl_3_) δ 7.44–7.29 (m, 5H), 7.24–7.15 (m, 3H), 7.07 (dd, *J* = 6.9, 2.3 Hz, 1H), 6.60 (d, *J* = 15.9 Hz, 1H), 6.22 (dd, *J* = 15.9, 7.1 Hz, 1H), 4.59–4.45 (m, 1H), 2.81–2.66 (m, 2H), 2.35 (s, 3H), 1.73 (s, 3H), 1.68 (s, 3H); ^13^C NMR (100 MHz, CDCl_3_) δ 144.6, 139.9, 138.0, 136.4, 132.5, 128.6, 128.6, 128.4, 128.3, 128.3, 128.2, 127.3, 123.7, 121.3, 83.6, 76.4, 42.1, 26.1, 24.0, 21.4; HRMS (ESI, *m*/*z*): calcd for C_22_H_23_ClO, [M+H]^+^: 339.1339, found: 339.1335.

(*Z*)-3-(chloro(phenyl)methylene)-2,2-dimethyl-5-((*E*)-2-methylstyryl) tetrahydrofuran (**4c**): 1-(Buta-1,3-dien-1-yl)-2-methylbenzene **2d** (0.30 mmol, 1.5 equiv.) with alkynol 2-methyl-4-phenylbut-3-yn-2-ol **1a** (0.20 mmol) gave compound **4c** (68%, 45.3 mg) as a yellow oil. ^1^H NMR (400 MHz, CDCl_3_) δ 7.49–7.32 (m, 6H), 7.22–7.15 (m, 3H), 6.87 (d, *J* = 15.7 Hz, 1H), 6.14 (dd, *J* = 15.7, 7.3 Hz, 1H), 4.60–4.53 (m, 1H), 2.79–2.68 (m, 2H), 2.36 (s, 3H), 1.76 (s, 3H), 1.71 (s, 3H); ^13^C NMR (100 MHz, CDCl_3_) δ 144.7, 140.0, 135.5, 135.5, 130.4, 130.3, 130.1, 128.4, 128.3, 128.3, 127.7, 126.1, 125.8, 121.3, 83.7, 76.8, 42.2, 26.2, 24.1, 19.8; HRMS (ESI, *m*/*z*): calcd for C_22_H_23_ClO, [M+H]^+^: 339.1337, found: 339.1335.

(*Z*)-5-((*E*)-4-(*tert*-Butyl)styryl)-3-(chloro(phenyl)methylene)-2,2-dimethyltetrahydro-furan (**4d**): 1-(Buta-1,3-dien-1-yl)-4-(*tert*-butyl)benzene **2e** (0.30 mmol, 1.5 equiv.) with alkynol 2-methyl-4-phenylbut-3-yn-2-ol **1a** (0.20 mmol) gave compound **4d** (73%, 55.3 mg) as a yellow oil. ^1^H NMR (400 MHz, CDCl_3_) δ 7.44 (dd, *J* = 8.5, 2.1 Hz, 4H), 7.35 (dd, *J* = 8.4, 6.6 Hz, 2H), 7.29 (dd, *J* = 7.8, 5.9 Hz, 3H), 6.71 (d, *J* = 16.0 Hz, 1H), 6.33 (dd, *J* = 16.0, 6.0 Hz, 1H), 4.66–4.59 (m, 1H), 2.71 (dd, *J* = 16.8, 10.6 Hz, 1H), 2.51 (dd, *J* = 16.8, 3.2 Hz, 1H), 1.62 (s, 6H), 1.38 (s, 9H); ^13^C NMR (100 MHz, CDCl_3_) δ 150.3, 136.8, 136.7, 133.0, 131.3, 131.2, 129.1, 128.6, 127.8, 127.8, 126.6, 125.1, 77.0, 69.1, 39.2, 34.6, 31.4, 28.6, 24.3; HRMS (ESI, *m*/*z*): calcd for C_25_H_29_ClO, [M+H]^+^: 381.1976, found: 381.1980.

(*Z*)-3-(chloro(phenyl)methylene)-5-((*E*)-3,5-difluorostyryl)-2,2-dimethyltetrahydro-furan (**4e**): 1-(Buta-1,3-dien-1-yl)-3,5-difluorobenzene **2f** (0.30 mmol, 1.5 equiv.) with alkynol 2-methyl-4-phenylbut-3-yn-2-ol **1a** (0.20 mmol) gave compound **4e** (60%, 43.0 mg) as a yellow oil. Yield: 60% (43 mg) as a yellow oil; ^1^H NMR (400 MHz, CDCl_3_) δ 7.44–7.30 (m, 6H), 6.88–6.76 (m, 2H), 6.72 (d, *J* = 16.1 Hz, 1H), 6.25 (dd, *J* = 16.1, 7.0 Hz, 1H), 4.57–4.47 (m, 1H), 2.78–2.65 (m, 2H), 1.73 (s, 3H), 1.68 (s, 3H); ^13^C NMR (100 MHz, CDCl_3_) δ 163.6 (d, *J* = 12.1 Hz), 161.3 (dd, *J* = 45.5, 12.0 Hz), 159.0 (d, *J* = 12.0 Hz), 144.4, 139.9, 131.0 (dd, *J* = 4.8, 2.2 Hz), 128.4 (dd, *J* = 5.8, 3.7 Hz), 123.9, 121.4, 120.6 (dd, *J* = 12.0, 3.9 Hz), 111.5 (dd, *J* = 21.4, 3.7 Hz), 104.0 (d, *J* = 25.8 Hz), 83.8, 42.0, 26.1, 24.0; ^19^F NMR (376 MHz, CDCl_3_) δ -110.56, -110.58, -113.59, -113.61; HRMS (ESI, *m*/*z*): calcd for C_21_H_19_ClF_2_O, [M+H]^+^: 361.0997, found: 361.0990.

(*Z*)-3-(chloro(phenyl)methylene)-5-((*E*)-4-chlorostyryl)-2,2-dimethyltetrahydro-furan (**4f**): 1-(Buta-1,3-dien-1-yl)-4-chlorobenzene **2g** (0.30 mmol, 1.5 equiv.) with alkynol 2-methyl-4-phenylbut-3-yn-2-ol **1a** (0.20 mmol) gave compound **4f** (66%, 47.0 mg) as a yellow oil. ^1^H NMR (400 MHz, CDCl_3_) δ 7.45–7.36 (m, 4H), 7.35–7.26 (m, 5H), 6.60 (d, *J* = 15.9 Hz, 1H), 6.21 (dd, *J* = 15.9, 7.0 Hz, 1H), 4.56–4.44 (m, 1H), 2.80–2.62 (m, 2H), 1.74 (s, 3H), 1.69 (s, 3H); ^13^C NMR (100 MHz, CDCl_3_) δ 144.4, 139.9, 134.9, 133.5, 131.1, 129.4, 128.7, 128.3, 127.7, 121.4, 83.7, 76.2, 42.0, 26.1, 24.0; HRMS (ESI, *m*/*z*): calcd for C_21_H_20_Cl_2_O, [M+H]^+^: 359.0795, found: 359.0789.

(*Z*)-3-(chloro(phenyl)methylene)-5-((*E*)-3-chlorostyryl)-2,2-dimethyltetrahydrofuran (**4g**): 1-(Buta-1,3-dien-1-yl)-3-chlorobenzene **2h** (0.30 mmol, 1.5 equiv.) with alkynol 2-methyl-4-phenylbut-3-yn-2-ol **1a** (0.20 mmol) gave compound **4g** (68%, 49.0 mg) as a yellow oil. ^1^H NMR (400 MHz, CDCl_3_) δ 7.44–7.33 (m, 6H), 7.24 (d, *J* = 3.6 Hz, 3H), 6.58 (d, *J* = 15.9 Hz, 1H), 6.25 (dd, *J* = 15.9, 6.8 Hz, 1H), 4.56–4.47 (m, 1H), 2.76–2.63 (m, 2H), 1.73 (s, 3H); ^13^C NMR (100 MHz, CDCl_3_) δ 144.3, 139.9, 138.4, 134.5, 130.9, 130.4, 129.8, 128.3, 127.8, 126.5, 124.7, 121.4, 83.8, 76.1, 42.0, 26.1, 24.0; HRMS (ESI, *m*/*z*): calcd for C_21_H_20_Cl_2_O, [M+H]^+^: 359.0797, found: 359.0789.

Methyl 4-((*E*)-2-((*Z*)-4-(chloro(phenyl)methylene)-5,5-dimethyltetrahydrofuran-2-yl)vinyl)benzoate (**4h**): Methyl (*E*)-4-(buta-1,3-dien-1-yl)benzoate **2i** (0.30 mmol, 1.5 equiv.) with alkynol 2-methyl-4-phenylbut-3-yn-2-ol **1a** (0.20 mmol) gave compound **4h** (62%, 47.0 mg) as a yellow oil. ^1^H NMR (400 MHz, CDCl_3_) δ 7.99 (d, *J* = 8.2 Hz, 2H), 7.46–7.31 (m, 7H), 6.67 (d, *J* = 15.9 Hz, 1H), 6.35 (dd, *J* = 15.9, 6.8 Hz, 1H), 4.58–4.50 (m 1H), 3.92 (s, 3H), 2.78–2.64 (m, 2H), 1.73 (s, 3H), 1.68 (s, 3H); ^13^C NMR (100 MHz, CDCl_3_) δ 166.8, 144.3, 140.9, 139.9, 131.5, 131.2, 129.9, 129.2, 128.3, 126.4, 121.5, 83.8, 52.1, 42.0, 26.1, 24.0; HRMS (ESI, *m*/*z*): calcd for C_23_H_23_ClO_3_, [M+H]^+^: 383.1410, found: 383.1408.

(*Z*)-3-(chloro(phenyl)methylene)-2,2-dimethyl-5-((*E*)-4-nitrostyryl)tetrahydrofuran (**4i**): 1-(Buta-1,3-dien-1-yl)-4-nitrobenzene **2j** (0.30 mmol, 1.5 equiv.) with alkynol 2-methyl-4-phenylbut-3-yn-2-ol **1a** (0.20 mmol) gave compound **4i** (60%, 46.0 mg) as a yellow oil. ^1^H NMR (400 MHz, CDCl_3_) δ 8.24–8.11 (m, 2H), 7.53–7.46 (m, 2H), 7.44–7.31 (m, 5H), 6.71 (d, *J* = 15.9 Hz, 1H), 6.43 (dd, *J* = 15.9, 6.5 Hz, 1H), 4.62–4.50 (m, 1H), 2.84–2.64 (m, 2H), 1.74 (s, 3H), 1.69 (s, 3H); ^13^C NMR (100 MHz, CDCl_3_) δ 147.1, 143.9, 142.9, 139.8, 133.8, 129.7, 128.4, 128.3, 128.3, 126.7, 123.9, 121.7, 84.4, 75.8, 41.9, 26.1, 24.0; HRMS (ESI, *m*/*z*): calcd for C_21_H_20_ClNO_3_, [M+H]^+^: 370.1206, found: 370.1204.

(*Z*)-3-(Chloro(phenyl)methylene)-2,2-dimethyl-5-((*E*)-2-(naphthalen-1-yl)vinyl)-tetrahydrofuran (**4j**): 1-(Buta-1,3-dien-1-yl)naphthalene **2k** (0.30 mmol, 1.5 equiv.) with alkynol 2-methyl-4-phenylbut-3-yn-2-ol **1a** (0.20 mmol) gave compound **4j** (58%, 43.0 mg) as a yellow solid, mp = 114.8–115.2 °C. ^1^H NMR (400 MHz, CDCl_3_) δ 7.80 (dd, *J* = 9.0, 6.8 Hz, 3H), 7.74 (d, *J* = 1.7 Hz, 1H), 7.61 (dd, *J* = 8.6, 1.7 Hz, 1H), 7.49–7.33 (m, 7H), 6.80 (d, *J* = 15.9 Hz, 1H), 6.37 (dd, *J* = 15.9, 7.0 Hz, 1H), 4.62–4.54 (m, 1H), 2.81–2.71 (m, 2H), 1.71 (s, 6H); ^13^C NMR (100 MHz, CDCl_3_) δ 144.6, 139.9, 133.9, 133.5, 133.1, 132.5, 129.1, 128.3, 128.3, 128.3, 128.2, 128.0, 127.7, 126.7, 126.3, 125.9, 123.6, 121.3, 83.7, 76.6, 42.2, 26.1, 24.0; HRMS (ESI, *m*/*z*): calcd for C_25_H_23_ClO, [M+H]^+^: 375.1338, found: 375.1335.

(*Z*)-3-(chloro(phenyl)methylene)-2,2-dimethyl-5-((*E*)-2-(thiophen-2-yl)vinyl)-tetrahydrofuran (**4k**): 2-(Buta-1,3-dien-1-yl)thiophene **2l** (0.30 mmol, 1.5 equiv.) with alkynol 2-methyl-4-phenylbut-3-yn-2-ol **1a** (0.20 mmol) gave compound **4k** (71%, 48.0 mg) as a yellow oil. ^1^H NMR (400 MHz, CDCl_3_) δ 7.44–7.43 (m, 2H), 7.42–7.32 (m, 3H), 7.30–7.21 (m, 2H), 7.04 (dd, *J* = 5.1, 3.8 Hz, 1H), 6.70 (d, *J* = 15.9 Hz, 1H), 6.30 (dd, *J* = 15.9, 7.1 Hz, 1H), 4.67–4.59 (m, 1H), 3.14 (dd, *J* = 16.2, 5.3 Hz, 1H), 2.83 (dd, *J* = 16.2, 10.1 Hz, 1H), 1.72 (s, 3H), 1.65 (s, 3H); ^13^C NMR (100 MHz, CDCl_3_) δ 144.6, 142.2, 136.5, 132.6, 128.7, 128.6, 127.9, 127.4, 126.8, 126.6, 126.2, 116.1, 84.5, 42.7, 26.2, 24.0; HRMS (ESI, *m*/*z*): calcd for C_19_H_19_ClOS, [M+H]^+^: 331.0821, found: 331.0819.

(*Z*)-3-(chloro(phenyl)methylene)-2,2-dimethyl-5-((*E*)-4-phenylbut-1-en-1-yl)tetrahydrofuran (**4l**): Hexa-3,5-dien-1-ylbenzene **2m** (0.30 mmol, 1.5 equiv.) with alkynol 2-methyl-4-phenylbut-3-yn-2-ol **1a** (0.20 mmol) gave compound **4l** (59%, 42.0 mg) as a yellow oil. ^1^H NMR (400 MHz, CDCl_3_) δ 7.44–7.28 (m, 7H), 7.22–7.15 (m, 3H), 5.85–5.75 (m, 1H), 5.58–5.50 (m, 1H), 4.34–4.26 (m, 1H), 2.74–2.66 (m, 2H), 2.64–2.54 (m, 2H), 2.42–2.32 (m, 2H), 1.69 (s, 3H), 1.63 (s, 3H); ^13^C NMR (100 MHz, CDCl_3_) δ 144.9, 141.7, 140.0, 133.7, 129.9, 128.4, 128.3, 128.2, 128.2, 125.9, 121.0, 83.4, 76.4, 42.0, 35.4, 34.2, 26.1, 23.9; HRMS (ESI, *m*/*z*): calcd for C_23_H_25_ClO, [M+H]^+^: 353.1403, found: 353.1402.

### 3.3. General Procedure for the Preparation of **5a**

A 25 mL round-bottomed flask was charged with **3a** (0.20 mmol) and 3-chloroperbenzoic acid (182.6 mg, 0.6 mmol, 3.0 equiv.). Then, the mixture was dissolved in DCM (5.0 mL) and stirred at r.t. for 8 h. The solvent was removed by rotary evaporation, and the residue was purified by flash column chromatography (PE/EA = 20:1) to give the product **5a** in a 75% yield.

(*E*)-3-(chloro(phenyl)methylene)-2,2-dimethyl-5-(3-phenyloxiran-2-yl)tetrahydrofuran (**5a**): Yield: 75% (49 mg) as a white solid, mp = 120.8–121.2 °C; ^1^H NMR (400 MHz, CDCl_3_) δ 7.45–7.32 (m, 9H), 7.29 (d, *J* = 1.9 Hz, 1H), 4.08–4.00 (m, 1H), 3.76 (d, *J* = 2.0 Hz, 1H), 3.19 (dd, *J* = 4.4, 2.0 Hz, 1H), 2.79–2.66 (m, 2H), 1.72 (s, 3H), 1.65 (s, 3H); ^13^C NMR (100 MHz, CDCl_3_) δ 143.4, 139.9, 136.7, 128.5, 128.4, 128.3, 128.3, 125.8, 121.7, 84.3, 62.9, 56.7, 37.3, 26.0, 24.1; HRMS (ESI, *m*/*z*): calcd for C_21_H_21_ClO_2_, [M+H]^+^: 326.1605, found: 326.1604.

### 3.4. General Procedure for the Preparation of **5b**

A 25 mL round-bottomed flask was charged with **3a** (0.20 mmol), NBS (53.4 mg, 0.3 mmol, 1.0 equiv.), and LiBr (34.7 mg, 0.4 mmol, 2.0 equiv.). Then, the mixture was dissolved in CH_3_CN (2.0 mL) and stirred at r.t. for 10 h. The solvent was removed by rotary evaporation, and the residue was purified by flash column chromatography (PE/EA = 15:1) to give the product **5b** in an 80% yield.

(*E*)-3-(Chloro(phenyl)methylene)-5-(1,2-dibromo-2-phenylethyl)-2,2-dimethyltetrahydrofuran (**5b**): Yield: 80% (75 mg) as a yellow oil; ^1^H NMR (400 MHz, CDCl_3_) δ 7.54–7.44 (m, 2H), 7.42–7.29 (m, 8H), 5.29 (d, *J* = 6.9 Hz, 1H), 4.65 (t, *J* = 6.8 Hz, 1H), 4.09–3.93 (m, 1H), 2.84–2.62 (m, 2H), 1.72 (s, 3H), 1.63 (s, 3H); ^13^C NMR (100 MHz, CDCl_3_) δ 142.7, 139.7, 137.7, 128.9 (d, *J* = 4.3 Hz), 128.4 (d, *J* = 3.6 Hz), 128.3, 128.2, 85.0, 61.5, 52.3, 38.6, 26.0, 24.3; HRMS (ESI, *m*/*z*): calcd for C_21_H_21_Br_2_ClO, [M+H]^+^: 470.5906, found: 470.5904.

### 3.5. General Procedure for the Preparation of **5c**

To a glass test tube (10 mL) was added **3a** (0.20 mmol, 1.0 equiv.), Pd/C (10 w%, 5.4 mg), and MeOH (1 mL). The tube was placed in a stainless-steel autoclave (Synthware, Beijing, China). After being sealed, the autoclave was purged three times with argon, and the final pressure of hydrogen was adjusted to 5 atm. The mixture was stirred at room temperature for 24 h, followed by release of the remaining gas. The reaction mixture was concentrated in vacuo. The residue was purified by flash chromatography on silica gel using PE/EA (20:1) as the eluent to give the product **5c** in a 72% yield.

(*E*)-3-(chloro(phenyl)methylene)-2,2-dimethyl-5-phenethyltetrahydrofuran (**5c**): Yield: 72% (47 mg) as a white solid, mp = 110.3–111.2 °C; ^1^H NMR (400 MHz, CDCl_3_) δ 7.42–7.35 (m, 4H), 7.34–7.27 (m, 3H), 7.19 (d, *J* = 8.2 Hz, 3H), 3.95–3.83 (m, 1H), 2.80–2.61 (m, 2H), 2.56 (dd, *J* = 15.6, 5.0 Hz, 1H), 2.40 (dd, *J* = 15.6, 10.1 Hz, 1H), 2.06–1.95 (m, 1H), 1.86–1.77 (m, 1H), 1.69 (s, 3H), 1.61 (s, 3H); ^13^C NMR (100 MHz, CDCl_3_) δ 145.1, 141.8, 140.1, 128.4, 128.3, 128.2, 128.1, 125.8, 120.9, 83.3, 41.3, 36.5, 32.1, 26.2, 23.9; HRMS (ESI, *m*/*z*): calcd for C_21_H_23_ClO, [M+H]^+^: 326.8607, found: 326.8609.

### 3.6. General Procedure for the Preparation of **7a**

A mixture of Pd(TFA)_2_ (10 mol %), CuCl_2_ (4 equiv.), DIPEA (80 mol%) and HOAc (2 mL) was added to a tube equipped with a stir bar. Then, propargyl alcohols (**6a**, 0.2 mmol), and alkene (**2a**, 0.3 mmol) were added to the tube under air and stirred at room temperature for 24 h. After the reaction was finished, the reaction was quenched by saturated aqueous NH_4_Cl and extracted with EtOAc three times. The combined organic layers were dried over anhydrous Na_2_SO_4_ and evaporated under a vacuum. The residue was purified by flash column chromatography on silica gel (eluting with petroleum ether/ethyl acetate) to afford the desired product **7a**.

(1*R*,2*S*,5*R*)-2-isopropyl-5-methylcyclohexyl 4-((*Z*)-chloro(2,2-dimethyl-5-((*E*)-styryl)dihydrofuran-3(2*H*)-ylidene)methyl)benzoate (**7a**): Yield: 65% (65 mg) as a yellow oil; ^1^H NMR (400 MHz, CDCl_3_) δ 8.06 (d, *J* = 8.1 Hz, 2H), 7.50 (d, *J* = 8.1 Hz, 2H), 7.38 (d, *J* = 7.3 Hz, 2H), 7.34–7.24 (m, 3H), 6.64 (d, *J* = 15.9, 1H), 6.22 (dd, *J* = 15.9, 6.8 Hz, 1H), 5.02–4.92 (m, 1H), 4.53 (q, *J* = 7.3 Hz, 1H), 2.78–2.65 (m, 2H), 2.15 (dt, *J* = 12.5, 3.8 Hz, 1H), 2.01–1.91 (m, 1H), 1.78 (t, *J* = 2.8 Hz, 1H), 1.74 (s, 3H), 1.68 (s, 3H), 1.63–1.54 (m, 2H), 1.20–1.08 (m, 2H), 1.04–0.97 (m, 1H), 0.95 (t, *J* = 6.2 Hz, 6H), 0.82 (d, *J* = 7.0 Hz, 3H); ^13^C NMR (100 MHz, CDCl_3_) δ 165.5, 146.0, 146.0, 144.0, 144.0, 136.4, 132.5, 132.5, 130.5, 130.5, 129.6, 129.6, 128.5, 128.3, 127.8, 126.6, 120.3, 83.8, 83.7, 76.4, 75.1, 47.3, 42.1, 42.1, 41.0, 34.3, 31.5, 26.6, 26.5, 26.1, 23.9, 23.7, 23.7, 22.0, 20.7, 16.6, 16.5; HRMS (ESI, *m*/*z*): calcd for C_32_H_39_ClO_3_, [M+H]^+^: 507.2488, found: 507.2486.

## 4. Conclusions

In summary, we have developed a robust synthetic strategy for the palladium-catalyzed cascade annulation reaction of buta-1,3-dienes with alkynols under mild reaction conditions. The catalytic strategy provides an effective and practical synthetic methodology for the assembly of diverse 3-methylenetetrahydrofurans with excellent functional group compatibility and good regioselectivity. A library of functional groups such as halogen atoms, aldehyde, nitro, ester, and several aromatic heterocycles was also nicely tolerated and provided the corresponding products in moderate to good yields. More importantly, the practicability is further verified by gram-scale experiments and synthetic transformation to access organic functional molecules. Studies of the detailed mechanism of this protocol and the asymmetric synthesis for the construction of chirality3-methylenetetrahydrofurans are ongoing in our laboratory.

## Figures and Tables

**Table 1:** Optimization of the reaction conditions ^a^.

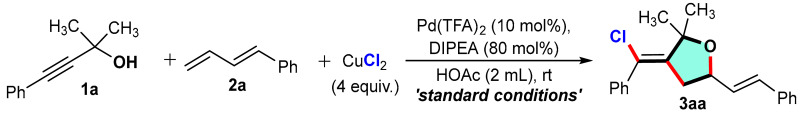			
**Entry**	**Variation from Standard Conditions**	**Yield** **^b^**	**Z/E** **^b^**
1	none	86 (82)	96:4
2	PdCl_2_ instead of Pd(TFA)_2_	57	96:4
3	Pd(OAc)_2_ instead of Pd(TFA)_2_	58	95:5
4	Pd(Py)_2_Cl_2_ instead of Pd(TFA)_2_	36	78:22
5	Pd(MeCN)_2_Cl_2_ instead of Pd(TFA)_2_	52	90:10
6	DMSO instead of HOAc	N.D.	-
7	MeCN instead of HOAc	N.D.	-
8	DCE instead of HOAc	38	94:6
9	Acetone instead of HOAc	43	83:17
10	Na_2_CO_3_ instead of DIPEA	54	90:10
11	DABCO instead of DIPEA	58	80:20
12	NEt_3_ instead of DIPEA	39	90:10
13	Cs_2_CO_3_ instead of DIPEA	trace	-
14	40 °C instead of rt	84	90:10
15	without Pd(TFA)_2_ or CuCl_2_	N.D.	-

^a^ Conditions: All reactions were performed with **1a** (0.10 mmol), **2a** (0.15 mmol), Pd(TFA)_2_ (10 mol%), CuCl_2_ (4 equiv.), DIPEA (80 mol%), and HOAc (2 mL) stirred at room temperature for 24 h. The Z/E ratio was determined by GC-MS analysis of the crude material. N.D. = not detected. ^b^ Determined by GC-MS using dodecane as the internal standard. The value in parentheses is the yield of the isolated product.

## Data Availability

Data are contained within the article and Supplementary Materials.

## Supplementary Materials

The following supporting information can be downloaded at: https://www.mdpi.com/article/10.3390/molecules30214244/s1, X-ray Crystallographic analysis of **3c** and **4j**; the NMR spectra and HRMS spectra of the catalytic products.
