# Supplementary material for: Assembly of Diverse 3-Methylenetetrahydrofurans via Palladium-Catalyzed Cascade Carboetherification of Buta-1,3-Dienes with Alkynols
Source: Molecules. 2025 Oct 31;30(21):4244. doi: 10.3390/molecules30214244 (PMC12608958; doi:10.3390/molecules30214244)
Supplement: Supplementary file 1 [file molecules-30-04244-s001.zip › molecules-3953566-supplementary.doc]

**Supporting Information**

Assembly of Diverse 3-Methylenetetrahydrofurans via Palladium-Catalyzed Cascade Carboetherification of Buta-1,3-Dienes with Alkynols

Yin-Long Lai ^1,2,^*, Yanan Niu ^3,†^, Kaitong Zeng ^3,†^, Huanfeng Jiang ^3^, Huishi Guo ^1,2,^*, Sheng-Ling Zhang ^1,2^ and Jianxiao Li ^3,4,^*

^1^	College of Chemistry and Civil Engineering, Shaoguan University, Shaoguan 512005, China;
zhangslisland@163.com

^2^	Guangdong Provincial Engineering Technology Research Center for Eco-Environmental Protection and
Solid Waste Utilization in Northern Guangdong, Shaoguan University, Shaoguan 512005, China

^3^	Key Laboratory of Functional Molecular Engineering of Guangdong Province, School of Chemistry and
Chemical Engineering, South China University of Technology, Guangzhou 510640, China;
ljx8872@126.com (Y.N.); 202330372451@mail.scut.edu.cn (K.Z.); jianghf@scut.edu.cn (H.J.)

^4^	Guangdong Provincial Key Laboratory of Utilization and Conservation of Food and Medicinal Resources in
Northern Region, Shaoguan University, Shaoguan 512005, China

*****	Correspondence: chemlaiyinlong@163.com (Y.-L.L.); gold0226@126.com (H.G.); cejxli@scut.edu.cn (J.L.)

^†^	These authors contributed equally to this work.

Table of Contents

X-ray Crystallographic analysis of **3c** and **4j**	S2

NMR Spectra for all the compounds	S11

HRMS Spectra for all the compounds	S59

**X-ray Crystallographic analysis for product 3c**

The CCDC number of the compound **3c** is 2448859

Crystal Data and Structure Refinement for Product **3c**

| Empirical formula | C_25_H_29_ClO |
| --- | --- |
| Formula weight | 380.93 |
| Temperature | 100 K |
| Wavelength | 0.71073 Å |
| Crystal system | triclinic |
| Space group | P-1 |
| Unit cell dimensions | a = 6.9186(8)Å, α= 75.207° |
|  | b= 11.8436(17) Å, β= 88.582 ^o^ |
|  | c= 14.0785 (2) Å, γ= 73.354^o^ |
| Density (calculated) | 1.186 |
| Absorption coefficient | 0.190 |
| F(000) | 408.0 |
| Crystal size | 0.15 × 0.08 × 0.05 |
| Theta range for data collection | 4.126 to 52.71 |
| Index ranges | -8≤h≤8, -14≤k≤13, -17≤l≤17 |
| Reflections collected | 13154 |
| Independent reflections | 4329 |
| Completeness to theta = 29.55° | 99.80% |
| Absorption correction | multi-scan |
| Refinement method | Full-matrix least-squares on F^2^ |
| Data/restraints/parameters | 4329/0/249 |
| Goodness-of-fit on F^2^ | 1.018 |
| Final R indices [I>2sigma(I)] | R_1_ = 0.0533, wR_2_ = 0.1061 |
| R indices (all data) | R_1_ = 0.0902, wR_2_ = 0.1252 |

**X-ray Crystallographic analysis for product 3j**

The CCDC number of the compound **3j** is 2448861

Crystal Data and Structure Refinement for Product 3j

| Empirical formula | C_25_H_23_ClO |
| --- | --- |
| Formula weight | 374.88 |
| Temperature | 106 K |
| Wavelength | 0.71073 Å |
| Crystal system | monoclinic |
| Space group | P2_1_ |
| Unit cell dimensions | a = 8.999(3) Å, *α*= 90° |
|  | b= 5.7493(14) Å, *β*= 91.955(10) ^o^ |
|  | c= 19.263(6) Å, *γ*= 90^o^ |
| Density (calculated) | 1.250 |
| Absorption coefficient | 0.203 |
| F(000) | 396.0 |
| Crystal size | 0.11 × 0.04 × 0.02 |
| Theta range for data collection | 4.232 to 52.744 |
| Index ranges | -11≤h≤11, -7≤k≤6, -24≤l≤17 |
| Reflections collected | 6061 |
| Independent reflections | 3500 |
| Completeness to theta = 29.55° | 99.80% |
| Absorption correction | multi-scan |
| Refinement method | Full-matrix least-squares on F^2^ |
| Data/restraints/parameters | 3500/535/337 |
| Goodness-of-fit on F^2^ | 1.074 |
| Final R indices [I>2sigma(I)] | R_1_ = 0.0854, wR_2_ = 0.1972 |
| R indices (all data) | R_1_ = 0.1483, wR_2_ = 0.2365 |

**^1^H and ^13^C NMR spectra of compounds**

**(*Z*)-3-(Chloro(phenyl)methylene)-2,2-dimethyl-5-((*E*)-styryl)tetrahydrofuran**

**^1^H NMR of 3a**

**^13^C NMR of 3a**

**(*Z*)-3-(Chloro(p-tolyl)methylene)-2,2-dimethyl-5-((*E*)-styryl)tetrahydrofuran**

**^1^H NMR of 3b**

**^13^C NMR of 3b**

**(*Z*)-3-((4-(*tert*-Butyl)phenyl)chloromethylene)-2,2-dimethyl-5-((*E*)-styryl)tetrahydrofuran**

**^1^H NMR of 3c**

**^13^C NMR of 3c**

**(*Z*)-3-(Chloro(4-chlorophenyl)methylene)-2,2-dimethyl-5-((*E*)-styryl)tetrahydrofuran**

**^1^H NMR of 3d**

**^13^C NMR of 3d**

**(*Z*)-3-((4-Bromophenyl)chloromethylene)-2,2-dimethyl-5-((*E*)-styryl)tetrahydrofuran**

**^1^H NMR of 3e**

**^13^C NMR of 3e**

**4-((*Z*)-Chloro(-2,2-dimethyl-5-((*E*)-styryl)dihydrofuran-3(2*H*)-ylidene)methyl)phenyl acetate**

**^1^H NMR of 3f**

**^13^C NMR of 3f**

**methyl 4-((*Z*)-Chloro(2,2-dimethyl-5-((*E*)-styryl)dihydrofuran-3(2*H*)-ylidene)methyl)benzoate**

**^1^H NMR of 3g**

**^13^C NMR of 3g**

**4-((*Z*)-Chloro(2,2-dimethyl-5-((*E*)-styryl)dihydrofuran-3(2*H*)-ylidene)methyl)benzaldehyde**

**^1^H NMR of 3h**

**^13^C NMR of 3h**

**(*Z*)-3-(chloro(4-(trifluoromethoxy)phenyl)methyl)-2,2-dimethyl-5-(*E*)-styryl)tetrahydrofuran**

**^1^H NMR of 3i**

**^13^C NMR of 3i**

**(*Z*)-3-(chloro(4-(trifluoromethyl)phenyl)methyl)-2,2-dimethyl-5-((*E*)-styryl)tetrahydrofuran**

**^1^H NMR of 3j**

**^13^C NMR of 3j**

**(*Z*)-3-(Chloro(3-methoxyphenyl)methylene)-2,2-dimethyl-5-((*E*)-styryl)tetrahydrofuran**

**^1^H NMR of 3k**

**^13^C NMR of 3k**

**(*Z*)-3-((3-Bromophenyl)chloromethylene)-2,2-dimethyl-5-((*E*)-styryl)tetrahydrofuran**

**^1^H NMR of 3l**

**^13^C NMR of 3l**

**(*Z*)-3-(Chloro(3-chlorophenyl)methylene)-2,2-dimethyl-5-((*E*)-styryl)tetrahydrofuran**

**^1^H NMR of 3m**

**^13^C NMR of 3m**

**(*Z*)-3-(Chloro(3-nitrophenyl)methylene)-2,2-dimethyl-5-((*E*)-styryl)tetrahydrofuran**

**^1^H NMR of 3n**

**^13^C NMR of 3n**

**(*Z*)-3-(Chloro(2-chlorophenyl)methylene)-2,2-dimethyl-5-((*E*)-styryl)tetrahydrofuran**

**^1^H NMR of 3o**

**^13^C NMR of 3o**

**(*Z*)-3-(Chloro(3,5-dimethylphenyl)methylene)-2,2-dimethyl-5-((*E*)-styryl)tetrahydrofuran**

**^1^H NMR of 3p**

**^13^C NMR of 3p**

**(*Z*)-3-((4-bromo-2-fluorophenyl)chloromethyl)-2,2-dimethyl-5-((*E*)-styryl)tetrahydrofuran**

**^1^H NMR of 3q**

**^13^C NMR of 3q**

**(*Z*)-3-(Chloro(naphthalen-1-yl)methylene)-2,2-dimethyl-5-((*E*)-styryl)tetrahydrofuran**

**^1^H NMR of 3r**

**^13^C NMR of 3r**

**(*Z*)-3-(Chloro(thiophen-2-yl)methylene)-2,2-dimethyl-5-((*E*)-styryl)tetrahydrofuran**

**^1^H NMR of 3s**

**^13^C NMR of 3s**

**(*Z*)-3-(Chloro(cyclopropyl)methylene)-2,2-dimethyl-5-((*E*)-styryl)tetrahydrofuran**

**^1^H NMR of 3t**

**^13^C NMR of 3t**

**(*Z*)-3-(1-Chlorobutylidene)-2,2-dimethyl-5-((*E*)-styryl)tetrahydrofuran**

**^1^H NMR of 3u**

**^13^C NMR of 3u**

**(*Z*)-3-(1-Chlorohexylidene)-2,2-dimethyl-5-((*E*)-styryl)tetrahydrofuran**

**^1^H NMR of 3v**

**^13^C NMR of 3v**

**(*Z*)-3-(1-Chlorononylidene)-2,2-dimethyl-5-((*E*)-styryl)tetrahydrofuran**

**^1^H NMR of 3w**

**^13^C NMR of 3w**

**(*Z*)-4-(Chloro(phenyl)methylene)-2-((*E*)-styryl)tetrahydrofuran**

**^1^H NMR of 3x**

**^13^C NMR of 3x**

**(*Z*)-3-(Chloro(phenyl)methylene)-2-methyl-5-((*E*)-styryl)tetrahydrofuran**

**^1^H NMR of 3y**

**^13^C NMR of 3y**

**(*Z*)-3-(Chloro(phenyl)methylene)-2-phenyl-5-((*E*)-styryl)tetrahydrofuran**

**^1^H NMR of 3aa**

**^13^C NMR of 3aa**

**(*Z*)-4-(Chloro(phenyl)methylene)-2-((*E*)-styryl)-1-oxaspiro[4.4]nonane**

**^1^H NMR of 3ab**

**^13^C NMR of 3ab**

**(*Z*)-4-(Chloro(phenyl)methylene)-2-((*E*)-styryl)-1-oxaspiro[4.5]decane**

**^1^H NMR of 3ac**

**^13^C NMR of 3ac**

**(*Z*)-3-(Chloro(naphthalen-1-yl)methylene)-2-methyl-5-((*E*)-styryl)tetrahydrofuran**

**^1^H NMR of 3ae**

**^13^C NMR of 3ae**

**(*Z*)-4-(Chloro(4-chlorophenyl)methylene)-2-((*E*)-styryl)-1-oxaspiro[4.4]nonane**

**^1^H NMR of 3af**

**^13^C NMR of 3af**

**(*Z*)-4-(Chloro(4-chlorophenyl)methylene)-2-((*E*)-styryl)-1-oxaspiro[4.5]decane**

**^1^H NMR of 3ag**

**^13^C NMR of 3ag**

**(*Z*)-4-((4-Bromophenyl)chloromethylene)-2-((*E*)-styryl)-1-oxaspiro[4.5]decane**

**^1^H NMR of 3ah**

**^13^C NMR of 3ah**

**(*Z*)-3-(Chloro(phenyl)methylene)-2,2-dimethyl-5-((*E*)-4-methylstyryl)tetrahydrofuran**

**^1^H NMR of 4a**

**^13^C NMR of 4a**

**(*Z*)-3-(Chloro(phenyl)methylene)-2,2-dimethyl-5-((*E*)-3-methylstyryl)tetrahydrofuran**

**^1^H NMR of 4b**

**^13^C NMR of 4b**

**(*Z*)-3-(Chloro(phenyl)methylene)-2,2-dimethyl-5-((*E*)-2-methylstyryl)tetrahydrofuran**

**^1^H NMR of 4c**

**^13^C NMR of 4c**

**(*Z*)-5-((*E*)-4-(*tert*-Butyl)styryl)-3-(chloro(phenyl)methylene)-2,2-dimethyltetrahydrofuran**

**^1^H NMR of 4d**

**^13^C NMR of 4d**

**(*Z*)-3-(Chloro(phenyl)methylene)-5-((*E*)-3,5-difluorostyryl)-2,2-dimethyltetrahydrofuran**

**^1^H NMR of 4e**

**^13^C NMR of 4e**

**(*Z*)-3-(Chloro(phenyl)methylene)-5-((*E*)-4-chlorostyryl)-2,2-dimethyltetrahydrofuran**

**^1^H NMR of 4f**

**^13^C NMR of 4f**

**(*Z*)-3-(Chloro(phenyl)methylene)-5-((*E*)-3-chlorostyryl)-2,2-dimethyltetrahydrofuran**

**^1^H NMR of 4g**

**^13^C NMR of 4g**

**Methyl 4-((*E*)-2-((*Z*)-4-(chloro(phenyl)methyl)-5,5-dimethyltetrahydrofuran-2-yl)benzoate**

**^1^H NMR of 4h**

**^13^C NMR of 4h**

**(*Z*)-3-(Chloro(phenyl)methylene)-2,2-dimethyl-5-((*E*)-4-nitrostyryl)tetrahydrofuran**

**^1^H NMR of 4i**

**^13^C NMR of 4i**

**(*Z*)-3-(Chloro(phenyl)methyl)-2,2-dimethyl-5-((*E*)-2-(naphthalen-1-yl)vinyl)tetrahydrofuran**

**^1^H NMR of 4j**

**^13^C NMR of 4j**

**(*Z*)-3-(Chloro(phenyl)methyl)-2,2-dimethyl-5-((*E*)-2-(thiophen-2-yl)vinyl)tetrahydrofuran**

**^1^H NMR of 4k**

**^13^C NMR of 4k**

**(*Z*)-3-(Chloro(phenyl)methylene)-2,2-dimethyl-5-((*E*)-4-phenylbut-1-en-1-yl)tetrahydrofuran**

**^1^H NMR of 4l**

**^13^C NMR of 4l**

**(*E*)-3-(Chloro(phenyl)methylene)-2,2-dimethyl-5-(3-phenyloxiran-2-yl)tetrahydrofuran**

**^1^H NMR of 5a**

**^13^C NMR of 5a**

**(*E*)-3-(Chloro(phenyl)methyl)-5-(1,2-dibromo-2-phenylethyl)-2,2-dimethyltetrahydrofuran**

**^1^H NMR of 5b**

**^13^C NMR of 5b**

**(*E*)-3-(Chloro(phenyl)methylene)-2,2-dimethyl-5-phenethyltetrahydrofuran**

**^1^H NMR of 5c**

**^13^C NMR of 5c**

**(1*R*,2*S*,5*R*)-2-Isopropyl-5-methylcyclohexyl 4-((*Z*)-chloro((*R*)-2,2-dimethyl-5-((*E*)-styryl)dihydrofuran-3(2*H*)-ylidene)methyl)benzoate**

**^1^H NMR of 7a**

**^13^C NMR of 7a**

**HRMS spectra for all compounds**
